# The C-Type Lectin Receptor Dectin-2 Is a Receptor for Aspergillus fumigatus Galactomannan

**DOI:** 10.1128/mbio.03184-22

**Published:** 2023-01-04

**Authors:** Jennifer L. Reedy, Arianne J. Crossen, Paige E. Negoro, Hannah Brown Harding, Rebecca A. Ward, Diego A. Vargas-Blanco, Kyle D. Timmer, Christopher M. Reardon, Kyle J. Basham, Michael K. Mansour, Marcel Wüthrich, Thierry Fontaine, Jean-Paul Latgé, Jatin M. Vyas

**Affiliations:** a Division of Infectious Diseases, Department of Medicine, Massachusetts General Hospital, Boston, Massachusetts, USA; b Harvard Medical School, Department of Medicine, Boston, Massachusetts, USA; c Department of Pediatrics, University of Wisconsin Medical School, University of Wisconsin Hospital and Clinics, Madison, Wisconsin, USA; d Institut Pasteur, Université de Paris, INRAE, USC2019, Unité Biologie et Pathogénicité Fongiques, Paris, France; e Institute of Molecular Biology and Biotechnology (IMBBFORTH), University of Crete, Heraklion, Greece; Duke University Hospital

**Keywords:** *Aspergillus fumigatus*, fungi, host-pathogen interactions, immune mechanisms, innate immunity, mycology

## Abstract

Aspergillus fumigatus is a ubiquitous environmental mold that causes significant mortality particularly among immunocompromised patients. The detection of the *Aspergillus*-derived carbohydrate galactomannan in patient serum and bronchoalveolar lavage fluid is the major biomarker used to detect A. fumigatus infection in clinical medicine. Despite the clinical relevance of this carbohydrate, we lack a fundamental understanding of how galactomannan is recognized by the immune system and its consequences. Galactomannan is composed of a linear mannan backbone with galactofuranose sidechains and is found both attached to the cell surface of *Aspergillus* and as a soluble carbohydrate in the extracellular milieu. In this study, we utilized fungal-like particles composed of highly purified *Aspergillus* galactomannan to identify a C-type lectin host receptor for this fungal carbohydrate. We identified a novel and specific interaction between *Aspergillus* galactomannan and the C-type lectin receptor Dectin-2. We demonstrate that galactomannan bound to Dectin-2 and induced Dectin-2-dependent signaling, including activation of spleen tyrosine kinase, gene transcription, and tumor necrosis factor alpha (TNF-α) production. Deficiency of Dectin-2 increased immune cell recruitment to the lungs but was dispensable for survival in a mouse model of pulmonary aspergillosis. Our results identify a novel interaction between galactomannan and Dectin-2 and demonstrate that Dectin-2 is a receptor for galactomannan, which leads to a proinflammatory immune response in the lung.

## INTRODUCTION

Aspergillus fumigatus is a saprophytic mold that causes a wide spectrum of clinical manifestations ranging from allergic disease to invasive infections, including pneumonia and disseminated disease. While the majority of patients that develop invasive aspergillosis are immunocompromised due to transplantation, inherited immune deficiencies, or use of immunosuppressant therapies, invasive aspergillosis also occurs in otherwise healthy individuals in association with preceding influenza or severe acute respiratory syndrome coronavirus 2 (SARS-CoV-2) infection (1–3). The mortality rate for invasive aspergillosis remains unacceptably high, with approximately 65% of patients succumbing to disease despite antifungal therapy (4), and is a major cause of morbidity, particularly among patients with compromised immune systems.

Humans are infected with A. fumigatus through inhalation of conidia deposited within small airways and alveoli. Conidia are eliminated in part by mechanical expulsion through the action of mucus and ciliated cells, as well as killing by macrophages and neutrophils (5, 6). Upon encountering a fungal pathogen, innate immune cells synchronously engage multiple fungal cell wall antigens through a diverse array of pattern recognition receptors, including Toll-like, scavenger, complement, and C-type lectin receptors (CLRs), and integrate those signals to generate a coordinated immune response (7–11). The majority of the fungal cell wall antigens are polysaccharides, as >90% of the fungal cell wall is composed of complex carbohydrates. The cell wall is commonly composed of an inner chitin and middle β-1,3 glucan layer with additional carbohydrate constituents like an outer mannan layer depending upon the genus of fungus (12–16). In A. fumigatus, the mannan content primarily exists within the galactomannan polymer. Although specific cell wall carbohydrate pattern recognition receptor interactions have been defined for a few cell wall polysaccharides, such as β-1,3 glucan and the CLR Dectin-1 (8, 17), we lack a fundamental understanding of the specific receptors that engage many other fungal carbohydrates and how these ligand-receptor cognate pairs drive the innate immune response.

The fungal cell wall is a highly dynamic structure that changes in both structure and composition depending on the morphological stage, environmental milieu, and upon engagement with immune cells (13, 18). Therefore, probing the role of a single carbohydrate entity using whole pathogens is challenging. Standard techniques have relied upon solubilized carbohydrates or genetic deletion mutants; however, these techniques have limitations, including pleiotropic changes that occur in genetic deletion strains, such as compensatory changes in cell wall composition or structure, growth defects, alterations in antigen exposure (19), and known differences in immune response to soluble versus particulate (or cell-bound) carbohydrates (20–22). Most fungal cell wall carbohydrates remain bound to the cell wall; however, galactomannan and β-1,3 glucan are unique, as they are both cell wall-associated and free/soluble carbohydrates. β-1,3 glucan has been extensively studied revealing differences in how soluble and cell wall β-1,3 glucan interact with immune cells (20–22). Cell wall-associated β-1,3 glucan signals via both the CLR Dectin-1 and complement receptor 3, whereas soluble β-1,3 glucan is Dectin-1 independent (20–22). Although both cell wall β-1,3 glucan and soluble β-1,3 glucan bind Dectin-1, only cell wall β-1,3 glucan activates signaling (20). In contrast, receptors such as TLRs signal in response to both soluble and cell-associated ligands. Activation of Dectin-1 requires receptor clustering into phagocytic synapses, which exclude regulatory phosphatases licensing activation of Dectin-1 (20, 23). Whether the difference in response to cell wall and soluble carbohydrates is unique to β-1,3 glucan and Dectin-1 or extends to other fungal carbohydrates and CLRs remains unknown. We hypothesize that the innate immune system distinguishes between soluble and cell-associated fungal carbohydrates to tailor the immune response. Most studies to date have been conducted using soluble galactomannan. Thus, we sought to characterize the immune response to cell-associated galactomannan to understand how innate immune cells interact with *Aspergillus* at the site of infection.

The carbohydrate galactomannan was identified approximately 40 years ago as an important clinical biomarker for the diagnosis of invasive aspergillosis, as it can be detected in serum and bronchial fluid from infected patients (24–27). However, the biological and immunological responses to this carbohydrate remain poorly defined. Galactomannan is composed of a linear mannan core composed of α-1,2-linked mannotetraose units attached via α-1,6-linkage with side changes composed of four to five β-1,5-linked galactofuranose residues (28, 29). Galactomannan can be found in both the conidial and mycelial stages of the *Aspergillus* life cycle. It is bound to the cell membrane through a glycosylphosphatidylinositol (GPI) anchor, covalently attached to β-1,3-glucan in the cell wall and also released as a soluble polysaccharide into the extracellular milieu (30, 31). While head-to-head comparisons of galactomannan from different strains of A. fumigatus has not been performed, the antibody-based detection of galactomannan as a biomarker for diagnosis of invasive *Aspergillus* infections provides evidence that galactomannan is a universal component of the *Aspergillus* cell wall. Most studies have utilized soluble galactomannan, since strains of *Aspergillus* that lack galactomannan have altered growth morphology and kinetics (32–36). Stimulation of peripheral blood mononuclear cells (PBMCs) with soluble galactomannan suppressed lipopolysaccharide (LPS)-induced cytokine production. Furthermore, an *Aspergillus* vaccine protection model demonstrated that treatment with soluble galactomannan induced a nonprotective Th2/Th17 immunity (37, 38). Dendritic cell (DC) and macrophages internalization of A. fumigatus conidia by the CLR dendritic cell-specific intercellular adhesion molecule-3-grabbing nonintegrin (DC-SIGN) and Pentraxin 3 could be inhibited through the addition of soluble galactomannan (39, 40), suggesting that both DC-SIGN and Pentraxin 3 could play a role in recognizing this carbohydrate. These observations indicated that *Aspergillus* may subvert the immune response by shedding galactomannan, but the innate immune response to cell-associated galactomannan has yet to be determined.

In this study, we identified Dectin-2 as a receptor for cell wall-associated galactomannan by using fungal-like particles (FLPs), a novel technique previously developed in our laboratory (41). To determine how the immune system recognizes galactomannan in the cell-associated state, we isolated galactomannan from A. fumigatus and created FLPs consisting of purified galactomannan. Using these particles to screen a library of CLR reporter cells, we identified Dectin-2 as a receptor for galactomannan. We confirmed the binding of Dectin-2 to galactomannan FLPs using a solubilized receptor and demonstrated that galactomannan FLPs stimulate production of tumor necrosis factor alpha (TNF-α) by murine macrophages in a Dectin-2-dependent manner. We also reveal that galactomannan induces Dectin-2-dependent differential gene expression. Furthermore, we determined that immunocompetent mice lacking Dectin-2 recruit increased numbers of immune cells to lungs compared with wild-type (WT) mice in a model of pulmonary aspergillosis, but Dectin-2 is dispensable for survival. Taken together, our results demonstrate a novel and important role for Dectin-2 in innate immune recognition of *Aspergillus* galactomannan.

## RESULTS

### Dectin-2 is a receptor for galactomannan.

**FIG 1:**
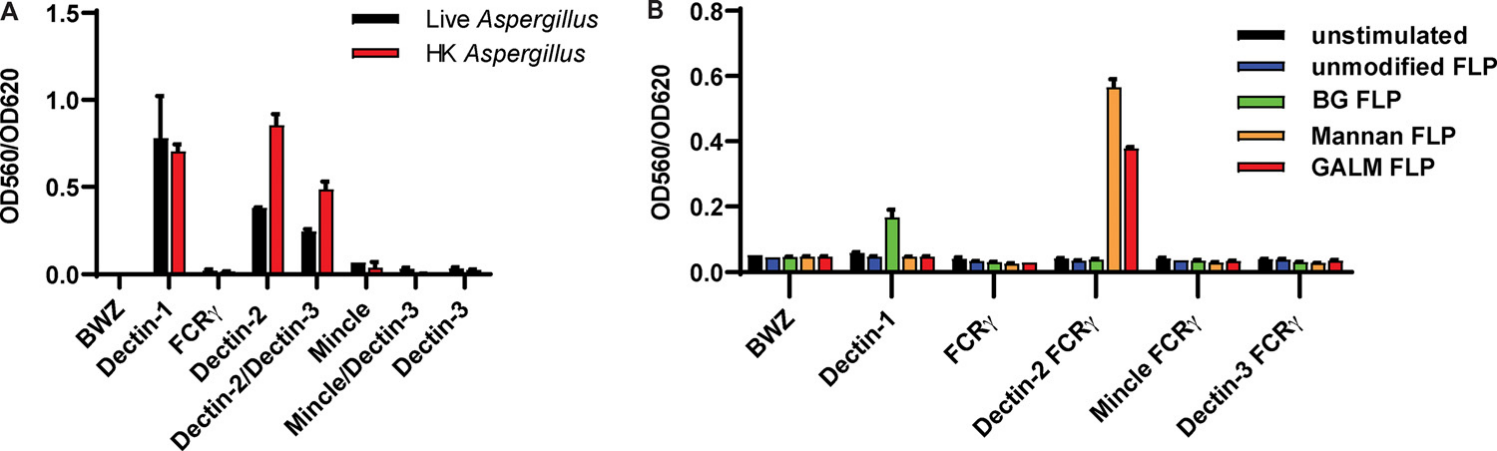
A. fumigatus and galactomannan activate Dectin-2-expressing CLR reporter cells. Reporter cells were stimulated for 18 h using either live or heat-killed germlings of A. fumigatus Af293 at a multiplicity of infection (MOI) of 20:1 (A) or unmodified FLPs, β-1,3 glucan (BG) FLPs, S. cerevisiae mannan FLPs, and A. fumigatus galactomannan (GALM) FLPs at an effector to target ratio of 30:1 (B). LacZ activity was measured in total cell lysates using CPRG as a substrate. Error bars represent the standard error of the mean (SEM) of duplicate wells, and each experiment was repeated three times.

Since CLRs are known to be involved in sensing of fungal pathogens and bind to carbohydrates, we examined if any of the known CLRs were potential receptors for galactomannan in the *Aspergillus* cell wall. We first screened a murine CLR library using A. fumigatus germlings to determine which CLRs were stimulated by the whole organism and confirm utility of the assay. T-cell hybridomas containing an NFAT-*lacZ* reporter were engineered to express individual or combinations of the following CLRs: Dectin-1, Dectin-2, Dectin-3 (MCL), or Mincle. Dectin-2, Dectin-3, and Mincle require interaction with the gamma subunit of the Fc gamma receptor (FcRγ) for both cell surface localization and signaling; thus, this protein was coexpressed. We stimulated the CLR reporter cells for 18 h with live and heat-killed *Aspergillus* germlings and observed robust activation of Dectin-1, Dectin-2, and Dectin-2/Dectin-3-coexpressing cells (Fig. 1A). Since cells that express Dectin-3 alone were not activated, the activation of the Dectin-2/Dectin-3-coexpressing cells is likely due to either activation of Dectin-2 alone or possible Dectin-2/Dectin-3 in combination (Fig. 1A). Activation of Dectin-1 is known to occur through binding of cell wall-associated β-1,3 glucan, but the Dectin-2 ligand on the surface of *Aspergillus* has not been established. We hypothesized that galactomannan could be the potential Dectin-2 ligand.

To dissect individual carbohydrate contributions to CLR reporter cell activation, we employed a technique developed in our laboratory for creating FLPs that consist of polystyrene beads coated with discrete, purified fungal carbohydrates (41). We have previously generated FLPs made from purified β-1,3 glucan and demonstrated that carbohydrate-coated FLPs mimic cell-associated or particulate carbohydrates. These β-1,3 glucan FLPs can activate Dectin-1 signaling and elicit the formation of a phagocytic synapse (23, 41, 42). Generation of galactomannan FLPs was performed using methods previously described for the creation of β-1,3 glucan and mannan FLPs (41). All generated FLPs underwent rigorous quality control using flow cytometry to demonstrate the stable attachment of carbohydrates to the FLP surface (see Fig. S1 in the supplemental material). As an added control, multiple independent purifications of the galactomannan carbohydrate were used for FLP creation for all experiments and yielded similar results. We stimulated the reporter cell library with unmodified beads, β-1,3 glucan FLP, mannan FLP, and galactomannan FLP. Activation of CLR signaling resulted in expression of a LacZ reporter and was quantified using a Chlorophenol red-β-D-galactopyranoside (CPRG) assay to measure β-galactosidase activity. As expected, β-1,3 glucan FLPs activated Dectin-1-expressing reporter cells, and S. cerevisiae mannan FLPs stimulated Dectin-2-expressing reporter cells. The galactomannan FLPs also stimulated Dectin-2 (Fig. 1B) and Dectin-2/Dectin-3-coexpressing cells (data not shown), suggesting that not only did galactomannan bind to Dectin-2 but also triggered signaling by the CLR. None of our FLPs stimulated Mincle or Dectin-3 signaling alone; however, these cells were activated by incubation with trehalose-6,6-dibehenate (TDB) (see Fig. S2 in the supplemental material), demonstrating that the receptors are functional and that absence of observed stimulation is due to lack of CLR activation by the galactomannan FLPs. These data suggested that Dectin-2, but not Dectin-3 or Mincle, is a receptor for *Aspergillus*-derived galactomannan.

10.1128/mbio.03184-22.1 FIG S1 Generation and validation of galactomannan FLP. (A) Galactomannan FLPs were created through adsorption or conjugation of purified *Aspergillus* galactomannan to the surface of amine-coated polystyrene beads. Flow cytometry comparing unmodified FLPs and galactomannan FLPs either unlabeled or incubated with anti-galactomannan antibody, followed by a secondary conjugated to Alexa Fluor 488 to detect galactomannan. Increased fluorescence indicates presence of galactomannan on the surface of the FLP. (B) Stability of carbohydrate attachment was assessed by incubating galactomannan FLPs with common laboratory detergents for 1 h, including Triton X-100, 3% SDS, and NP40. FLPs were then washed and analyzed by flow cytometry. For both conjugated and adsorbed FLPs, detergent washing did not disrupt surface association of the galactomannan to the FLP core. (C) FLPs were then boiled for 1 h in 3% SDS and assessed for carbohydrate stability. Under these stringent conditions, there was slight reduction in surface association of galactomannan from adsorbed FLP but not conjugated. (D) Immunofluorescence of galactomannan FLP. Unmodified FLP, β-1,3 glucan FLP, and galactomannan FLP were labeled with anti-galactomannan antibody followed by secondary labelled with AF488 and analyzed using spinning disc confocal microscopy. Arrows indicating galactomannan FLP. Scale bar, 5 nm. Download FIG S1, PDF file, 0.2 MB. Copyright © 2023 Reedy et al. 2023 Reedy et al. https://creativecommons.org/licenses/by/4.0/ This content is distributed under the terms of the Creative Commons Attribution 4.0 International license.

10.1128/mbio.03184-22.2 FIG S2 CLR reporter cell activation using CLR control agonists. Reporter cell lines were stimulated for 18 h with 10 μg/mL TDB (Mincle, Dectin-3 agonist), 10 μg/mL Furfurman (Dectin-2 agonist), or 100 μg/mL Zymosan (Dectin-1, Dectin-2 agonist). β-galactosidase activity was measured in total cell lysates using CPRG as a substrate. Error bars represent the SEM of duplicate wells, and each experiment was repeated three times. As expected, TDB stimulated β-galactosidase production in cells containing either Mincle or Dectin-3, Furfurman stimulated Dectin-2 containing cells, and Zymosan stimulates Dectin-1- or Dectin-2-containing cell lines. Of note, the intrinsic β-galactosidase-producing capacity of each cell line differs; therefore, cross-cell line comparisons regarding the strength of the signal does not necessarily indicate differences in binding or signaling efficiency to a ligand. Download FIG S2, PDF file, 0.4 MB. Copyright © 2023 Reedy et al. 2023 Reedy et al. https://creativecommons.org/licenses/by/4.0/ This content is distributed under the terms of the Creative Commons Attribution 4.0 International license.

### Soluble Dectin-2 binds to galactomannan FLPs and *Aspergillus* swollen conidia and hyphae.

**FIG 2:**
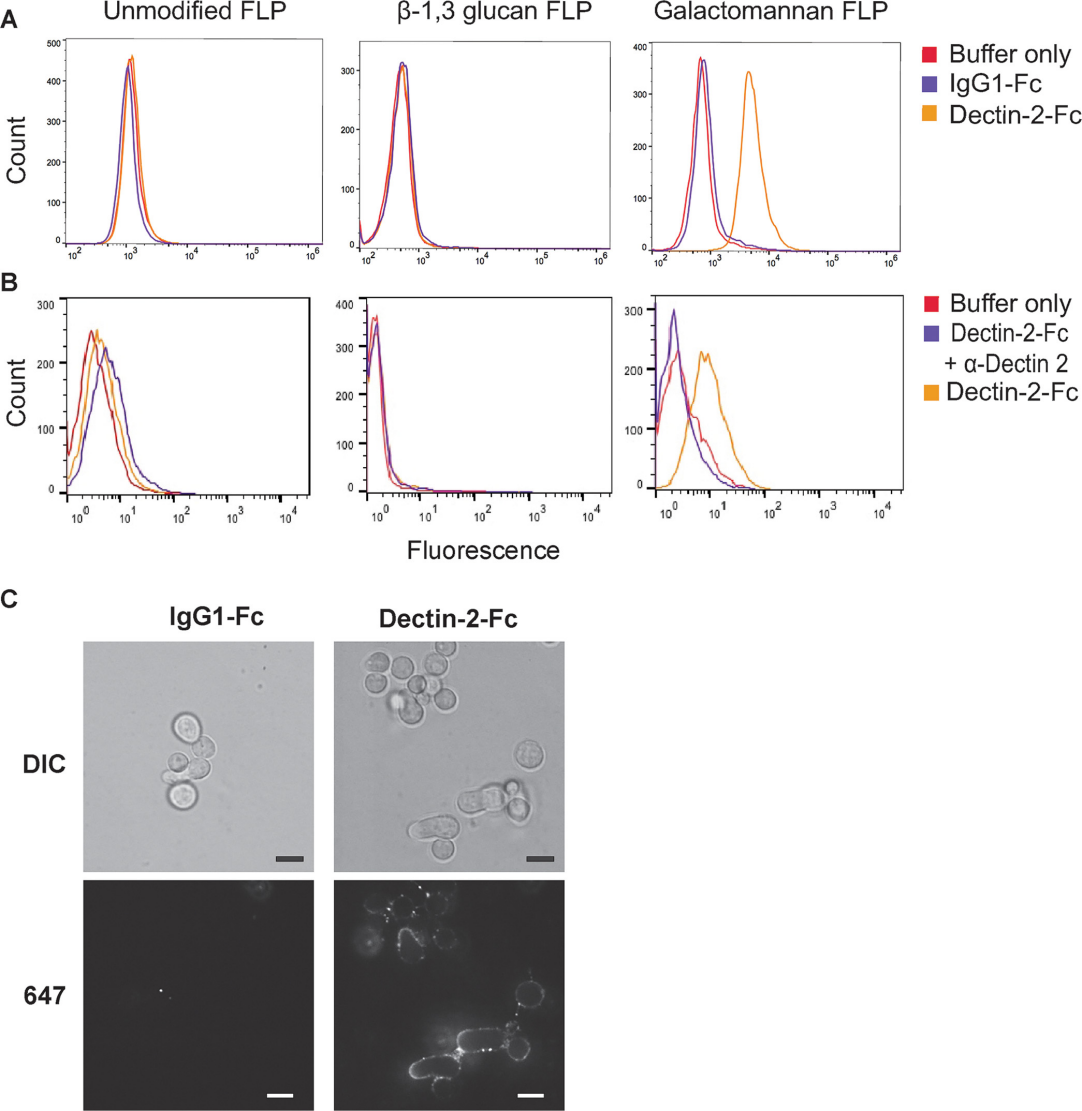
Recombinant Dectin-2 Fc protein binds galactomannan FLPs and A. fumigatus. (A) Unmodified FLPs, β-1,3 glucan FLPs, and galactomannan FLPs were incubated with soluble recombinant murine Dectin-2-human IgG1 Fc protein conjugate, control IgG1-Fc protein, or in buffer only, incubated with anti-human IgG1 conjugated to Alexa Fluor 488 and analyzed by flow cytometry. Dectin-2-Fc bound to galactomannan FLPs but not to either unmodified or β-1,3 glucan FLPs. (B) FLPs were incubated for 2 h in binding buffer alone or containing Dectin-2-Fc protein or Dectin-2-Fc preincubated for 30 min with a Dectin-2-neutralizing antibody. Dectin-2-neutralizing antibody disrupted the association of Dectin-2-Fc with galactomannan FLPs. (C) A. fumigatus CEA10 swollen conidia and early germlings were incubated with Dectin-2-Fc protein or control protein, IgG1-Fc, followed by incubation with anti-human IgG Fc conjugated to Alexa Fluor 647 and analyzed by confocal microscopy. Dectin-2-Fc protein, but not control IgG1-Fc, bound intensely to the early hyphal structure and to swollen conidia.

After identifying Dectin-2 as a receptor for galactomannan using the CLR reporter cell screen, we next assessed whether Dectin-2 binds directly to galactomannan FLP. To evaluate this, we used soluble murine Dectin-2-human IgG1 Fc fusion protein and a control human IgG1 Fc protein (lacking Dectin-2). Flow cytometry using an anti-human IgG1 Fc antibody and an AF488-labeled secondary antibody was used to detect adherence of Dectin-2-Fc to the surface of FLP (Fig. 2A). Dectin-2-Fc was specifically bound to both *Aspergillus* galactomannan FLPs and *Saccharomyces* mannan FLPs but did not bind to either unmodified or β-1,3 glucan FLPs. As expected, human IgG1 Fc protein did not bind to any FLP. As an additional control to demonstrate that binding to the galactomannan FLPs was Dectin-2 specific, we incubated the Dectin-2-Fc protein with anti-Dectin-2 neutralizing antibody or isotype control for 30 min prior to incubation with galactomannan FLPs. The Dectin-2-neutralizing antibody, but not the isotype control, potently blocked binding of Dectin-2-Fc to galactomannan FLP, demonstrating a specific interaction (Fig. 2B).

To demonstrate that Dectin-2 also binds to the *Aspergillus* cell wall, we incubated *Aspergillus* swollen conidia and early germlings with either Dectin-2-Fc or IgG1-Fc followed by an AF 647-labeled secondary antibody. Imaging of the fungi using confocal microscopy demonstrated binding of soluble Dectin-2-Fc, but not IgG1-Fc, to both hyphae and swollen conidia with the highest intensity of binding to hyphal tips (Fig. 2C). Resting conidia did not bind the Dectin-2-Fc as would be expected due to shielding of the cell wall by the melanin and Rod A layers (data not shown).

### Galactomannan FLPs induce Dectin-2-dependent Syk activation.

**FIG 3:**
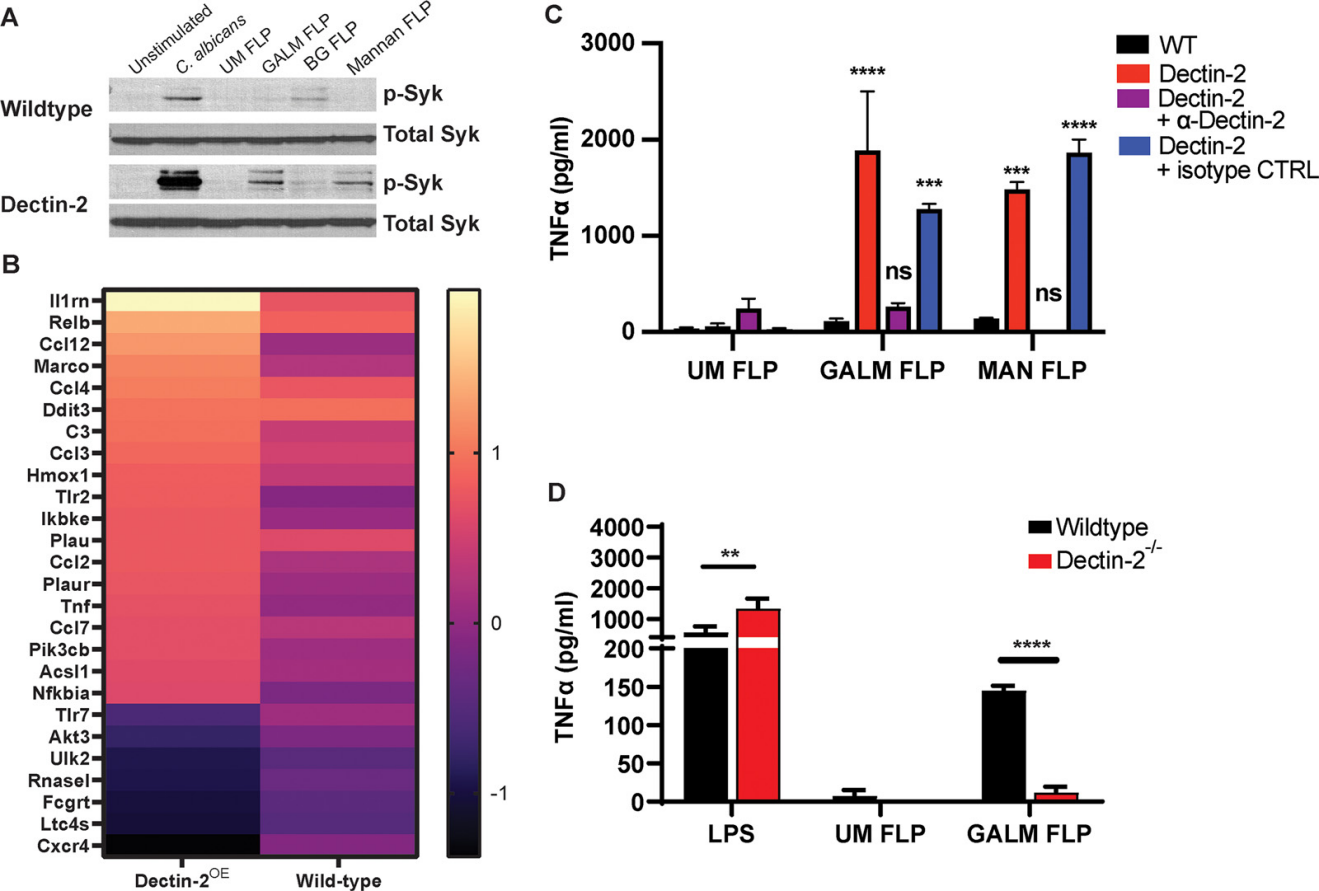
Galactomannan FLPs activate Dectin-2-dependent signaling in macrophages. (A) Immunoblot of immortalized wild-type C57BL/6 macrophages or C57BL/6 macrophages expressing Dectin-2 stimulated with live C. albicans (SC5314), unmodified FLPs (UM FLP), galactomannan FLPs (GALM FLP), β-1,3 glucan FLPs (BG FLP), or mannan FLPs at an MOI of 10:1 for 1 h at 37°C and probed for both total Syk and phosphorylated (active) Syk. Overexpression of Dectin-2 increased phosphorylation of Syk in response to C. albicans, galactomannan and mannan FLPs, but not to unmodified or β-1,3 glucan FLPs. (B) Heatmap displaying the genes differentially expressed in Dectin-2^OE^ macrophages stimulated with GALM-FLP compared with unstimulated. The heatmap depicts the log_2_ fold change in Dectin-2^OE^ cells and wild-type cells demonstrating a higher degree of change for most genes in the Dectin-2^OE^ cells compared to those of the WT. (C) Immortalized wild-type C57BL/6 macrophages or C57BL/6 macrophages expressing Dectin-2 were stimulated overnight with unmodified (UM), galactomannan (GALM), or mannan (MAN) FLPs at a target to effector ratio of 10:1. Prior to FLP stimulation, macrophages were pretreated for 30 min with either a Dectin-2-neutralizing antibody or an isotype control antibody as indicated. Supernatants were analyzed for TNF-α using ELISA. Both galactomannan and mannan FLPs induced robust TNF-α production by macrophages expressing Dectin-2, which was blocked by Dectin-2-neutralizing antibody but not the isotype control. The significance indicated is in relation to the wild-type cell stimulated with the same FLPs. (D) BMDMs from wild-type C57BL/6 and Dectin-2^−/−^ mice were stimulated with LPS (500 ng/mL), and unmodified (UM) or galactomannan (GALM) FLPs at an effector to target ration of 20:1 for 18 h. Supernatants were analyzed for TNF-α using ELISA. Dectin-2^−/−^ BMDM produced less TNF-α when stimulated with galactomannan FLP compared with wild-type BMDM. Error bars represent standard error of three biological replicates, statistics were calculated with two-way analysis of variance (ANOVA) using PRISM 9 software (not significant, ns; ***, *P* < 0.001; ****, *P* < 0.0001).

The CLR reporter assay and binding studies demonstrated that Dectin-2 is capable of binding *Aspergillus* galactomannan. Next, we investigated whether this binding results in downstream signaling. Activation of Dectin-2 through ligand binding triggers phosphorylation of the ITAM motif of FcRγ and subsequent signaling through Syk. FcRγ is also required for localization of Dectin-2 to the cell surface (43). Macrophages express Dectin-2; however, immortalized C57BL/6 murine macrophages have low levels of Dectin-2 surface expression at baseline (data not shown). Therefore, we transduced macrophages with lentivirus containing the murine Dectin-2 receptor. As macrophages produce abundant FcRγ, we did not observe any difference in Dectin-2 surface expression between macrophages transduced with only Dectin-2 or those transduced with both Dectin-2 and FcRγ. To determine if galactomannan is sufficient to trigger Dectin-2-dependent Syk activation, we stimulated macrophages with galactomannan FLPs for 1 h as well as unmodified FLPs, β-1,3 glucan FLPs, mannan FLPs, and Candida albicans. We used C. albicans as a positive control in these experiments since it is a strong activator of Syk phosphorylation in wild-type macrophages. Lysates from stimulated cells were immunoblotted for phosphorylated and total Syk. As expected, we observed phosphorylation of Syk when wild-type macrophages were stimulated by Candida albicans and β-1,3 glucan FLPs (positive controls for Syk stimulation) (Fig. 3A). There was increased phosphorylation of Syk in response to galactomannan and in Dectin-2 expressing macrophages demonstrating that *Aspergillus* galactomannan is sufficient to drive Dectin-2-dependent Syk phosphorylation (Fig. 3A).

### Galactomannan induces Dectin-2-dependent differential gene expression.

To determine whether galactomannan can induce Dectin-2-dependent transcriptional changes, we stimulated immortalized C57BL/6 macrophages (wild type) or C57BL/6 macrophages expressing Dectin-2 (Dectin-2^OE^) for 6 h with galactomannan FLP and identified genes that were differentially expressed compared with unstimulated wild-type or unstimulated Dectin-2^OE^ cells using NanoString. RNA samples were obtained from three biological replicates for each condition (unstimulated or galactomannan FLP stimulated) and were analyzed using the nCounter host response panel. Genes were considered to be significant if they demonstrated at least a 1.5-fold change in expression (log_2_ fold change [log_2_ FC], >0.58 or <−0.58) and had an adjusted *P* value of <0.5 using the Benjamini-Hochberg method for estimating false discovery rate (FDR). A total of 19 genes that were significantly induced in Dectin-2^OE^ macrophages in response to galactomannan FLPs compared with only 2 in wild-type macrophages. Seven genes had significantly reduced expression in the Dectin-2^OE^ macrophages when stimulated with galactomannan FLPs (Fig. 3B; see also Table S1 in the supplemental material). The majority of differentially expressed genes in the Dectin-2^OE^ macrophages had similar directional change in wild-type macrophages but to a lesser degree. We compared the fold change between wild-type and Dectin-2^OE^ macrophages and found that the fold changes were significantly different for at least 14 of the genes. The differentially expressed genes induced by galactomannan FLP in the Dectin-2^OE^ macrophages included those involved in TNF signaling (TNF, CCL2, PIK3CB), NF-κB signaling (RELB, CCL4, CCL2, TNF, NFKBIA), and cellular migration (CCL12, CCL4, CCL3, CCL2, TNF, CCL7) (Table S1).

10.1128/mbio.03184-22.6 TABLE S1 Differentially expressed genes. Download Table S1, DOCX file, 0.02 MB. Copyright © 2023 Reedy et al. 2023 Reedy et al. https://creativecommons.org/licenses/by/4.0/ This content is distributed under the terms of the Creative Commons Attribution 4.0 International license.

### Dectin-2 mediates TNF-α production by murine macrophages in response to both A. fumigatus and galactomannan FLPs.

Since we demonstrated that galactomannan is sufficient to activate Dectin-2-dependent Syk signaling, and Dectin-2 induced transcriptional responses, including induction of TNF signaling, we next interrogated if galactomannan stimulation of Dectin-2 is both sufficient and necessary for TNF-α production. To determine if galactomannan stimulation of Dectin-2 is sufficient to trigger cytokine secretion, Dectin-2-expressing murine macrophages were stimulated overnight with unmodified FLPs, β-1,3 glucan FLPs, A. fumigatus galactomannan FLPs, or S. cerevisiae mannan FLPs (positive control), and TNF-α production was measured by enzyme-linked immunosorbent assay (ELISA). Galactomannan and mannan FLPs enhanced TNF-α production in Dectin-2-expressing macrophages (Fig. 3C). To demonstrate that this effect was specific to Dectin-2, we preincubated the macrophages with a Dectin-2-neutralizing antibody or an isotype control for 30 min prior to stimulation with FLPs. TNF-α production was blocked by the Dectin-2-specific antibody but not by the isotype control, demonstrating a specific role for Dectin-2 in mediating the TNF-α production in response to galactomannan (Fig. 3C).

To examine if Dectin-2 is required for primary bone marrow-derived macrophages (BMDMs) to respond to galactomannan FLPs, primary BMDM from wild-type or Dectin-2-deficient mice were stimulated by galactomannan FLPs, unmodified FLPs (negative control), or LPS (positive control). There was near complete reduction of TNF-α production in response to galactomannan FLPs (Fig. 3D), indicating that galactomannan triggers the secretion of proinflammatory cytokines in BMDMs in a Dectin-2-dependent manner.

### Deletion of Dectin-2 results in increased recruitment of immune cells but does not alter fungal burden.

**FIG 4:**
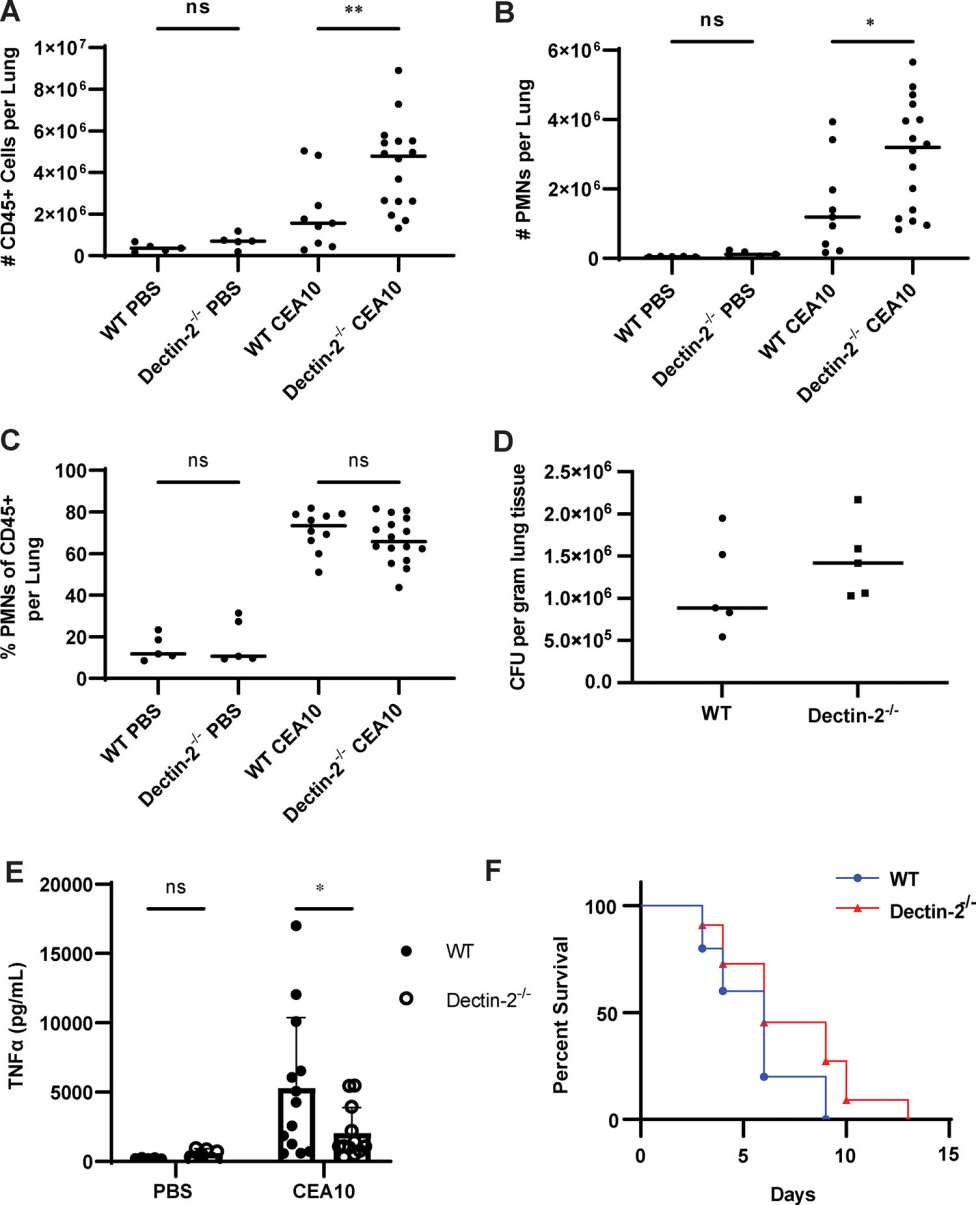
Deficiency of Dectin-2 leads to increased immune cell recruitment and decreased pulmonary TNF-α levels but is dispensable for survival. (A to E) Immunocompetent wild-type and Dectin-2^−/−^ mice were infected via oropharyngeal inhalation of 4 × 10^7^ conidia of A. fumigatus CEA10. Lung tissue was harvested 48 h postinfection and digested to obtain a single cell suspension that was then analyzed by flow cytometry. Dectin-2-infected mice had (A) increased infiltration of live CD45^+^ cells compared with that of the wild type. There was a significant increase in the total number of (B) neutrophils (7-AAD^lo^, CD45^+^, CD90.2^−^, CD19^−^, SiglecF^−^, Ly6G^+^) in Dectin-2^−/−^ mice compared with wild type. However, there was no difference in the percentage of CD45^+^ cells that were neutrophils (C). The data shown are representative data from a single experiment. Each point represents an individual animal and horizontal lines represent the median. One-way ANOVA was performed using PRISM 9 software to determine statistical significance with Šídák’s multiple comparison test (not significant, ns; *, *P* < 0.05; **, *P* < 0.01; ***, *P* < 0.001; ****, *P* < 0.0001). (D) Immunocompetent wild-type and Dectin-2^−/−^ mice were infected with 4 × 10^7^ CEA10. After 48 h, lungs were extracted and fungal burden was quantified using CFU. There was no difference in fungal burden between wild-type and Dectin-2^−/−^ mice. Each point represents an individual animal, and horizontal lines represent the median fungal burden in the lungs. Data shown are from a single experiment consisting of 5 infected and 1 PBS control per mouse strain. Each experiment was repeated at least twice, and concordant results were obtained in each experiment. (E) Immunocompetent wild-type and Dectin-2^−/−^ mice were infected with 4 × 10^7^ CEA10 or PBS, and after 48 h, lungs were harvested and homogenized for cytokine analysis. Infected Dectin-2^−/−^ mice had significantly less TNF-α compared with wild-type mice, and there was no difference in TNF-α between PBS-stimulated mice. Each point represents an individual animal, and boxes represent the mean with error bars representing the standard deviation. Two-way ANOVA was performed using PRISM 9 software to determine statistical significance with Šídák’s multiple comparison test (not significant, ns; *, *P* < 0.05; **, *P* < 0.01; ***, *P* < 0.001; ****, *P* < 0.0001). (F) Wild-type and Dectin-2^−/−^ mice were immunosuppressed with corticosteroids and infected intranasally with 2.5 × 10^4^ conidia of A. fumigatus strain CEA10. There was no significant difference in survival between wild-type and Dectin-2^−/−^ mice as determined based upon Kaplan-Meyer and Cox analysis using PRISM 9 software.

We sought to determine if the immune cell influx in response to infection was altered in the absence of Dectin-2. Since treatment with steroids affects immune cell function, we chose to perform this experiment in an immunocompetent rather than a steroid-induced immunosuppression mouse model. Immunocompetent mice were administered either A. fumigatus strain CEA10 or phosphate-buffered saline (PBS) via oropharyngeal aspiration. After 48 h of infection, lungs were harvested, digested, and the resulting single cell suspension was enriched for leukocytes using a Percoll gradient. A multichannel flow cytometry experiment was performed to allow simultaneous assessment of multiple cell lines, including neutrophils, eosinophils, dendritic cells (including cDC1, cDC2, monocyte-derived DCs, and plasmacytoid dendritic cells [pDCs]), NK cells, and monocytes. All infected mice demonstrated a marked increase in CD45^+^ cellular infiltrates compared to those of the PBS control mice. We infected mice with 1 × 10^7^, 2 × 10^7^, and 4 × 10^7^ conidia of CEA10 A. fumigatus and noted a dose-dependent increase in immune cell infiltrate. At doses higher than 4 × 10^7^, most mice succumbed within 24 to 48 h, presumably due to an exuberant inflammatory response. Infected Dectin-2^−/−^ mice recruited more CD45^+^ cells to the lungs than did infected wild-type mice (Fig. 4A). Additionally, Dectin-2^−/−^-infected lungs had a significant increase in neutrophils compared to the wild type (Fig. 4B, Fig. S3). We did not find any consistent differences in the lung populations of eosinophil, dendritic cell, monocyte, or NK cell populations between wild-type or Dectin-2^−/−^-infected animals (see Fig. S4 in the supplemental material). Notably, the proportion of the CD45^+^ cellular infiltrate comprised of neutrophils (Fig. 4C) was not significantly different between infected wild-type and Dectin-2^−/−^ mice, suggesting that there is increased immune cell recruitment to the lungs during infection in Dectin-2^−/−^ mice but no change in the overall proportion of neutrophil populations. Our results suggest that Dectin-2 is involved in the coordination of inflammatory responses in this airway model of infection.

10.1128/mbio.03184-22.3 FIG S3 Flow cytometry gating strategy for lung immunophenotyping to identify neutrophils. (A) Samples were first gated based upon FSC-A versus SSC-A to separate debris from cells. (B and C) Cells were then gated using FSC-H versus FSC-A (B) followed by SSC-H versus SSC-A (C) to select for single cells. (D) Single cells were then gated using 7-AAD to identify live (7-AAD^lo^) versus dead cells (7-AAD^high^) and CD45-BV605 to identify live CD45^+^ cells (7-AAD^lo^ CD45^+^). (E) The population of live CD45^+^ cells was then separated based on CD90.2-BV786 and CD19-PE-Dazzle to separate T-cells (CD90.2^+^ CD19^−^) and B-cells (CD90.2^−^ CD19^+^). (F) The CD90.2^−^ CD19^−^ cell population was then further separated using SiglecF-APC and Ly6G-AF488. Neutrophils were identified as SiglecF^lo^ Ly6G^high^ population. Download FIG S3, PDF file, 0.6 MB. Copyright © 2023 Reedy et al. 2023 Reedy et al. https://creativecommons.org/licenses/by/4.0/ This content is distributed under the terms of the Creative Commons Attribution 4.0 International license.

10.1128/mbio.03184-22.4 FIG S4 Immunophenotyping and fungal burden in WT versus Dectin-2^−/−^ mice. (A) Immunophenotyping of pulmonary leukocytes in and Dectin-2^−/−^ mice infected with PBS (mock infection) or 4 × 10^7^ conidia of A. fumigatus strain CEA10. Each point represents a single animal and the data shown are from a single experiment but are representative of other experimental replicates. There was no difference seen in immune cell infiltrates between WT and Dectin-2^−/−^ mice with mock PBS infection. While there was a trend towards increased numbers of immune cells in the examined cell populations in the Dectin-2^−/−^ mice compared with WT when infected with CEA10, this difference only reached the level of significance for eosinophils, CD103^+^ CD11b^−^ cDCs and pDCs in one out of three replicate experiments. (B) Fungal burden was measured at 48 h using qPCR to quantify the burden of fungal DNA in the lungs. There was no significant difference between WT and Dectin-2^−/−^ mice. Download FIG S4, PDF file, 0.5 MB. Copyright © 2023 Reedy et al. 2023 Reedy et al. https://creativecommons.org/licenses/by/4.0/ This content is distributed under the terms of the Creative Commons Attribution 4.0 International license.

To determine whether the increased influx of CD45^+^ cells could be the result of difference in fungal burden between wild-type and Dectin-2-deficient mice, we quantified fungal burden in the lungs of mice infected with 4 × 10^7^ conidia at 48 h postinfection using quantitative PCR (qPCR) to detect and quantify *Aspergillus* DNA (44). A. fumigatus DNA was detected in all samples from infected mice but not in PBS controls. There was no difference in *Aspergillus* DNA between wild-type and Dectin-2-deficient mice (see Fig. S4 in the supplemental material). CFU assays were also performed as a confirmatory test to ensure that there was no difference in live *Aspergillus* fungal burden since fungal DNA testing cannot distinguish between live versus dead organisms. Lungs from both wild-type and Dectin-2-deficient mice still contained live A. fumigatus at 48 h, and there was no difference in CFU between strains (Fig. 4D). Taken together, these results suggest that the enhanced immune cell infiltration in Dectin-2-deficient mice is not due to a difference in fungal burden.

### Infected Dectin-2 mice have decreased pulmonary TNF-α compared with wild-type mice.

To determine if there were differences in the pulmonary cytokine milieu at 48 h that could account for the increased inflammatory cell recruitment to the lungs of Dectin-2^−/−^ mice, we measured pulmonary cytokines using lung homogenates from wild-type and Dectin-2^−/−^ mice infected with either PBS or 4 × 10^7^ conidia of A. fumigatus *CEA10* for 48 h. We observed decreased TNF-α in the lungs of infected Dectin-2^−/−^ mice compared with wild-type mice (Fig. 4E). There was no difference in pulmonary concentrations of CXCL2, KC, gamma interferon (IFN-γ), interleukin-6 (IL-6), IL-1β, IL-12(p70), IP-10, MCP-1, or RANTES between wild-type and Dectin-2^−/−^ mice (see Fig. S5B in the supplemental material). However, there was an increase in baseline levels of granulocyte-macrophage colony-stimulating factor (GM-CSF), IFN-α, IFN-β, and IL-10 in the Dectin-2^−/−^ mice treated with PBS compared with those of wild-type mice but no significant difference in the infected mice (Fig. S5A), suggesting that the baseline inflammatory milieu in the Dectin-2^−/−^ mice may be different compared with wild-type mice.

10.1128/mbio.03184-22.5 FIG S5 Pulmonary cytokine analysis comparing wild-type (WT, closed circles) and Dectin-2^−/−^ mice (open circles) infected for 48 h with either PBS or A. fumigatus strain CEA10. Lung homogenates were analyzed using Biolegend LEGENDplex or traditional ELISA. (A) Several cytokines, including GM-CSF, IFN-β, IFN-α, and IL-10, were increased in Dectin-2^−/−^ compared with those of wild-type mice at 48 h in the PBS controls; however, there was no difference in cytokine levels between CEA10-infected mice. (B) There was no significant difference in the cytokine levels between wild-type and Dectin-2^−/−^ mice stimulated with either PBS or A. fumigatus for CXCL2, KC, IFNγ, IL-6, IL-1β, IL-12(p70), IP-10, MCP-1, and RANTES. Data shown are the composite data from three independent experiments. Each point represents a single mouse. Bars represent the mean cytokine level, and error bars represent the standard deviation, statistics were calculated using two-way ANOVA with Šídák’s multiple comparison test (not significant, ns; *, *P* < 0.05; **, *P* < 0.01). Download FIG S5, PDF file, 0.5 MB. Copyright © 2023 Reedy et al. 2023 Reedy et al. https://creativecommons.org/licenses/by/4.0/ This content is distributed under the terms of the Creative Commons Attribution 4.0 International license.

### Dectin-2 is dispensable for survival in a murine model of infection.

Our data suggest that Dectin-2 is a receptor for A. fumigatus galactomannan and that recognition of galactomannan by Dectin-2 results in Syk phosphorylation, cytokine production, and increased inflammatory cell recruitment in Dectin-2^−/−^ mice infected with A. fumigatus. To determine if this pathway is required for survival during *Aspergillus* infection, we intranasally infected corticosteroid immunosuppressed Dectin-2^−/−^ and wild-type C57BL/6 mice with A. fumigatus (CEA10 strain) and monitored the mice for signs of infection. There was no difference in survival between wild-type and Dectin-2-deficient mice, suggesting that in this model, Dectin-2 is dispensable for survival (Fig. 4F).

## DISCUSSION

Galactomannan is a clinically important polysaccharide released into patients’ blood and bronchial lavage fluid and used to diagnose invasive aspergillosis infections. However, we do not understand the immunological implications of the wide-scale release of this carbohydrate into patient tissue or the role that it plays in mediating direct immunological interactions between A. fumigatus and innate immune cells at the site of infection. Here, we utilized FLPs coated with purified A. fumigatus galactomannan to identify Dectin-2 as a receptor for galactomannan. Our results demonstrate that cell-associated galactomannan binds Dectin-2-inducing phosphorylation of Syk, transcriptional changes, and TNF-α production.

Although BMDMs from Dectin-2-deficient mice had decreased cytokine production in response to galactomannan FLPs, there was no difference in overall survival of immunosuppressed Dectin-2-deficient mice compared with that of wild-type mice when challenged intranasally with A. fumigatus. There is significant redundancy in CLR signaling; therefore, it is possible that the lack of Dectin-2 is compensated for through Dectin-1 or other CLR-ligand interactions in the context of the whole organism. Additionally, Pentraxin (PTX3) and DC-SIGN are alternative galactomannan receptors, since binding of both receptors to A. fumigatus can be blocked by the addition of soluble galactomannan (39, 40). Thus, alternative mechanisms of galactomannan recognition in macrophages and dendritic cells could also compensate for the absence of Dectin-2. However, publications have suggested a critical role for Dectin-2 in the immune response to A. fumigatus (45, 46). A recent case report identified a patient without previously recognized immunodeficiency that developed invasive aspergillosis and was found to have a mutation in Dectin-2 that led to decreased responsiveness to *Aspergillus* (45). Previous work has also demonstrated a role for Dectin-2 in mediating pDC responses to A. fumigatus (46). These observations, coupled with this study, suggest that while Dectin-2 may not be required for survival in a murine model, it may contribute to human disease through modulating interactions between discrete immune cell populations.

Interestingly, immunocompetent mice that lacked Dectin-2 have increased recruitment of immune cells into the lung tissue upon infection with *Aspergillus*, which is in sharp contrast to galactosaminogalactan (GAG) from A. fumigatus, which led to a reduction of neutrophil infiltration (47). The difference in inflammatory cell infiltrates was not due to difference in the fungal burden within the lungs, suggesting that it is a direct result of Dectin-2 deficiency. We anticipated that the absence of Dectin-2 would result in decreased pulmonary inflammation but surprisingly observed increased immune cell recruitment. Interestingly, analysis of pulmonary cytokines demonstrated decreased pulmonary TNF-α in the infected Dectin-2^−/−^ mice compared with that in the wild type. This correlates with our *in vitro* data demonstrating that galactomannan-induced Dectin-2 signaling in macrophages leads to proinflammatory TNF-α release. As an effector cytokine that is important for activation of macrophages and dendritic cells as well as phagocyte activation, it is possible that the enhanced immune cell recruitment is to compensate for decreased activity of macrophages or other early cell types. At earlier time points, it is possible that there could be differences in fungal burden that is driving the immune cell influx seen at 48 h; however, we did not observe a difference in fungal burden at 48 h. There was no significant change in the pulmonary levels of either KC or CXCL2, which are known neutrophil chemoattractants. Of note, there were observed cytokine difference in our PBS mock-infected mice with higher levels of type I IFNs (IFN-α, IFN-β), IL-10, and GM-CSF in the Dectin-2^−/−^ mice compared with the wild type, but there was no difference in the cytokine levels in the A. fumigatus-infected mice. There was no difference in the lung immune cell populations in wild-type mice compared to Dectin-2^−/−^ mice with PBS, suggesting that these differences did not influence the innate immune compartments analyzed. There are multiple potential hypotheses to explain the increased inflammatory response in Dectin-2-deficient mice, which will provide fertile ground for future studies. In the absence of Dectin-2, expression of Dectin-1 and/or other C-type lectin receptors may be upregulated, and thus, these receptors may play a more substantial role in driving the immune response. Additionally, Dectin-2 is expressed not only by macrophages but also by many immune cells and other lung resident cells, including epithelial cells. Thus, a cell type other than macrophages may be responsible for mediating the difference in immune cell influx that we observed. Interestingly, the increased immune cell influx observed in Dectin-2-deficient mice did not affect fungal burden, suggesting that the increased immune cells infiltrate did not result in more rapid clearance of infection. Whether the increased inflammatory influx seen in Dectin-2-deficient mice could have detrimental effects on the host, such as increasing lung damage during infection, is unknown.

Our current work addresses the mechanism by which cell wall-associated galactomannan is recognized by Dectin-2. Galactomannan and β-1,3 glucan are both fungal cell wall carbohydrates that exist in both soluble and cell wall-associated states. β-1,3 glucan interacts with the immune system differently depending upon its form. Whether galactomannan-induced immune activation is triggered only at sites of infection or more systemically and how this impacts the outcome of infection remain critically important questions. Whether circulating soluble galactomannan can stimulate recognition by Dectin-2 will be the subject of future studies. Dectin-2 has also been implicated in allergic disease (48–51), suggesting that galactomannan from *Aspergillus* could play a role in stimulating allergic manifestations to this pathogen. Overall, our data demonstrate that galactomannan is recognized by Dectin-2 and lays the exciting groundwork for dissecting the role of galactomannan in important clinical disease.

## MATERIALS AND METHODS

### Animals.

Mice were maintained in specific-pathogen-free barrier facilities at Massachusetts General Hospital (MGH) (Boston, MA) according to Institutional Animal Care and Use Committee (IACUC) guidelines. Wild-type C57BL/6 mice were obtained from Jackson Laboratories (Bar Harbor, ME) and Dectin-2^−/−^ mice (52) were from the University of Wisconsin—Madison. All mice used for bone marrow harvests and *in vivo* experiments were 8 to 20 weeks of age and were cohoused prior to experiments. Mice were both age- and sex-matched for each experiment and an equal distribution of male and female mice used.

### Cell lines and culture.

Immortalized murine C57BL/6 bone marrow-derived macrophages (BMDM) were a gift from Doug Golenbock (University of Massachusetts Medical School, Worcester, MA). Macrophages were cultured in complete RPMI medium (cRMPI) (RPMI 1650, l-glutamine, 10% heat-inactivated fetal bovine serum [FBS] [Gibco, Thermo Fisher Scientific, Rockford, IL], 1% penicillin/streptomycin, 1% HEPES buffer, and 50 μM β-mercaptoethanol). Puromycin and blasticidin were added to a final concentration of 5 μg/mL as needed for the selection of transduced cells. Primary BMDMs from C57BL/6 and Dectin-2^−/−^ mice were harvested as previously described (42). After harvest, primary BMDMs were grown for 7 days in complete RPMI medium containing 20 ng/mL of recombinant macrophage colony-stimulating factor (M-CSF) (Peprotech, Rocky Hill, NJ) prior to use in assays. HEK293T cells were purchased from the ATCC (American Type Cell Collection, Manassas, VA) and were grown in complete DMEM (DMEM, l-glutamine, 10% heat-inactivated FBS, 1% penicillin/streptomycin, 1% HEPES buffer). All cell lines were grown at 37°C in the presence of 5% CO_2_.

### Fungal culture.

A. fumigatus strains Af293 and CEA10 were grown at 37°C for 5 days on Sabouraud’s dextrose agar (SBD) (Difco, Fisher Scientific, Rockford, IL) or for 3 days on glucose minimal media slants (GMM), respectively. Slants were seeded from frozen stocks of conidia maintained at −80°C. To harvest conidia, a sterile solution of deionized water (Milli-Q; Millipore Sigma, Burlington, MA) containing 0.01% Tween 20 was added to each slant, and spores were liberated using gentle surface agitation with a sterile swab. The spore solution was passed through a 40-μm cell strainer to separate hyphal debris. Spores were washed three times with sterile PBS and counted on a LUNA automated cell counter (Logos Biosystems, Annandale, VA) and diluted appropriately. To generate A. fumigatus germlings, conidia were incubated in cRPMI media at 37°C for 4 to 6 h and visually inspected for the presence of emerging germ tubes. Germlings were then washed, counted, and resuspended in PBS at the desired inoculum. For heat-killing, A. fumigatus germlings were incubated at 95°C for 30 min.

### Purification of galactomannan.

The galactomannan was isolated as lipo-galactomannan from the cellular membrane of A. fumigatus mycelium that had been grown in SBD medium using previously described methods (30). The lipid moiety was removed by nitrous deamination, and the polysaccharide moiety was purified by gel filtration chromatography on a Superdex 75 column (10/300 GL, GE-Healthcare) (30). Three independent preparations of galactomannan were used to create FLP, and multiple batches were used per experiment to confirm that results were not batch dependent.

### FLP binding assays.

FLPs were blocked overnight in PBS containing 2% bovine serum albumin (BSA). FLPs were washed and resuspended in 100 μL of binding buffer (20 mM Tris-HCL, pH 7.4, 150 mM NaCl, 10 mM CaCl_2_, 0.05% Tween 20). Dectin-2(murine)-IgG1 Fc (human) fusion protein (ENZO Life Sciences, Inc, Farmingdale, NY) or IgG1 Fc (human) protein (Thermo Fisher Scientific, Rockford, IL) was added to a final concentration of 10 μg/mL, and samples were incubated with gentle mixing for 2 h at room temperature. Following washing, samples were resuspended in binding buffer containing a 1:100 dilution of mouse anti-human-IgG1-Fc conjugated to Alexa Fluor 488 (Thermo Fisher Scientific, Rockford, IL; A-10631) and incubated for 30 min at room temperature in the dark. FLPs were washed with binding buffer, resuspended in fluorescence-activated cell sorter (FACS) buffer (PBS containing 2% BSA), examined on a BD FACSCalibur or BD FACSCelesta flow cytometer (BD, Franklin Lakes, NJ), and data analyzed using FlowJo 10. For neutralizing antibody experiments, mouse anti-Dectin-2 antibody (R&D Systems, MAB1525) was preincubated with the Dectin-2-Fc fusion protein at a concentration of 50 mg/mL for 30 min prior to adding the mixture to FLPs. For additional materials and methods, please see Text S1.

10.1128/mbio.03184-22.1 TEXT S1 Supplemental Materials and Methods. Download TEXT S1, PDF file, 0.1 MB. Copyright © 2023 Reedy et al. 2023 Reedy et al. https://creativecommons.org/licenses/by/4.0/ This content is distributed under the terms of the Creative Commons Attribution 4.0 International license.
