# Supplementary figures and images for: The C-Type Lectin Receptor Dectin-2 Is a Receptor for Aspergillus fumigatus Galactomannan
Source: mBio. 2023 Jan 4;14(1):e03184-22. doi: 10.1128/mbio.03184-22 (PMC9973300; doi:10.1128/mbio.03184-22)

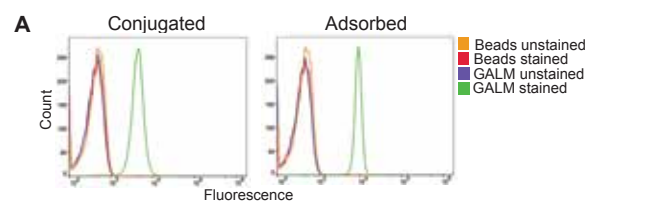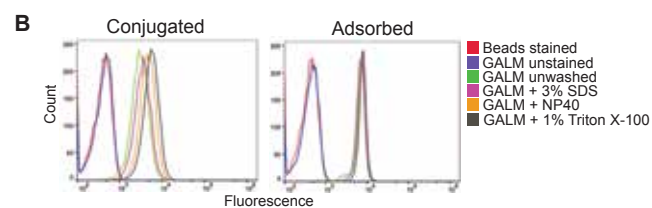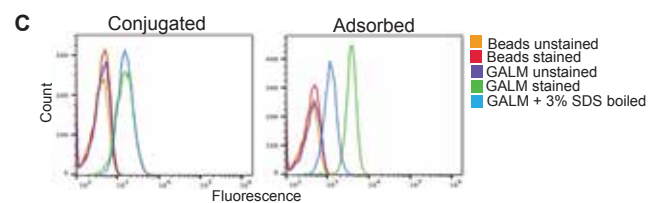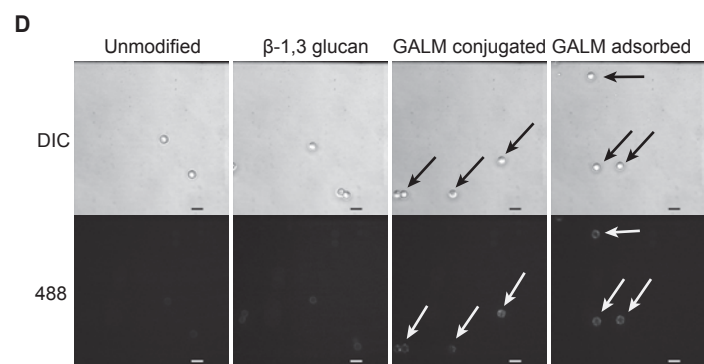

Supplement: FIG S1 [file mbio.03184-22-s0003.pdf]

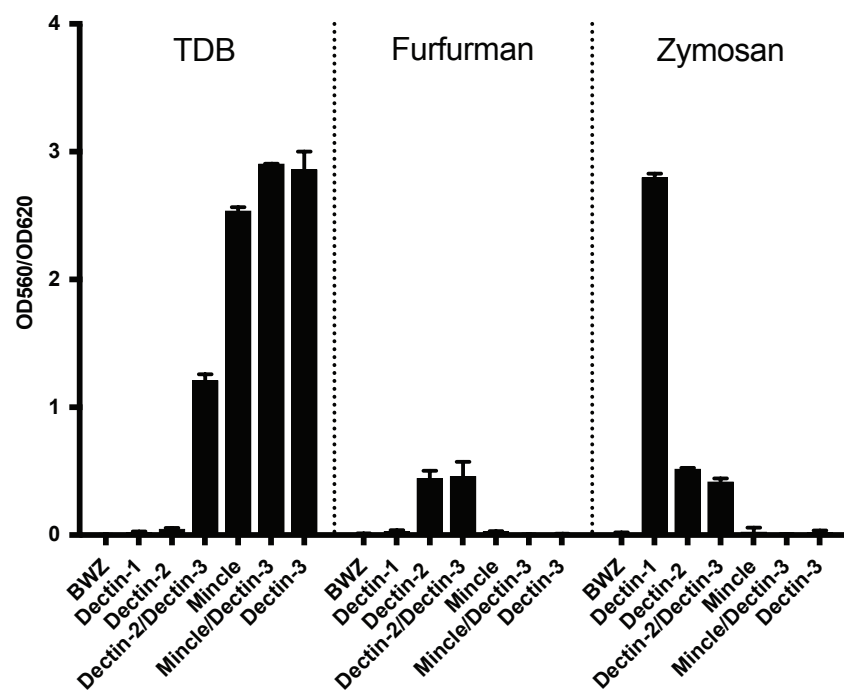

Supplemental Figure S2

Supplement: FIG S2 [file mbio.03184-22-s0004.pdf]

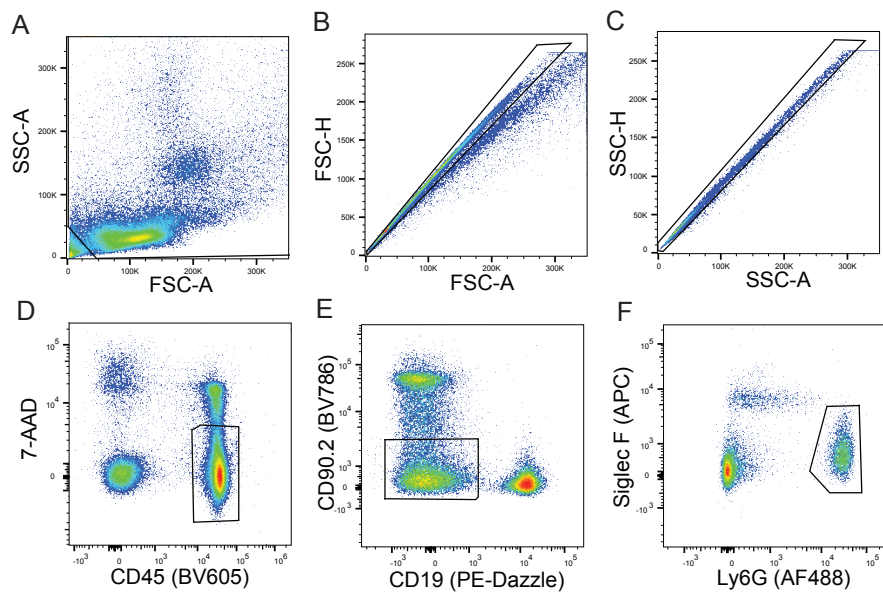

**Supplemental Figure S3**

Supplement: FIG S3 [file mbio.03184-22-s0005.pdf]

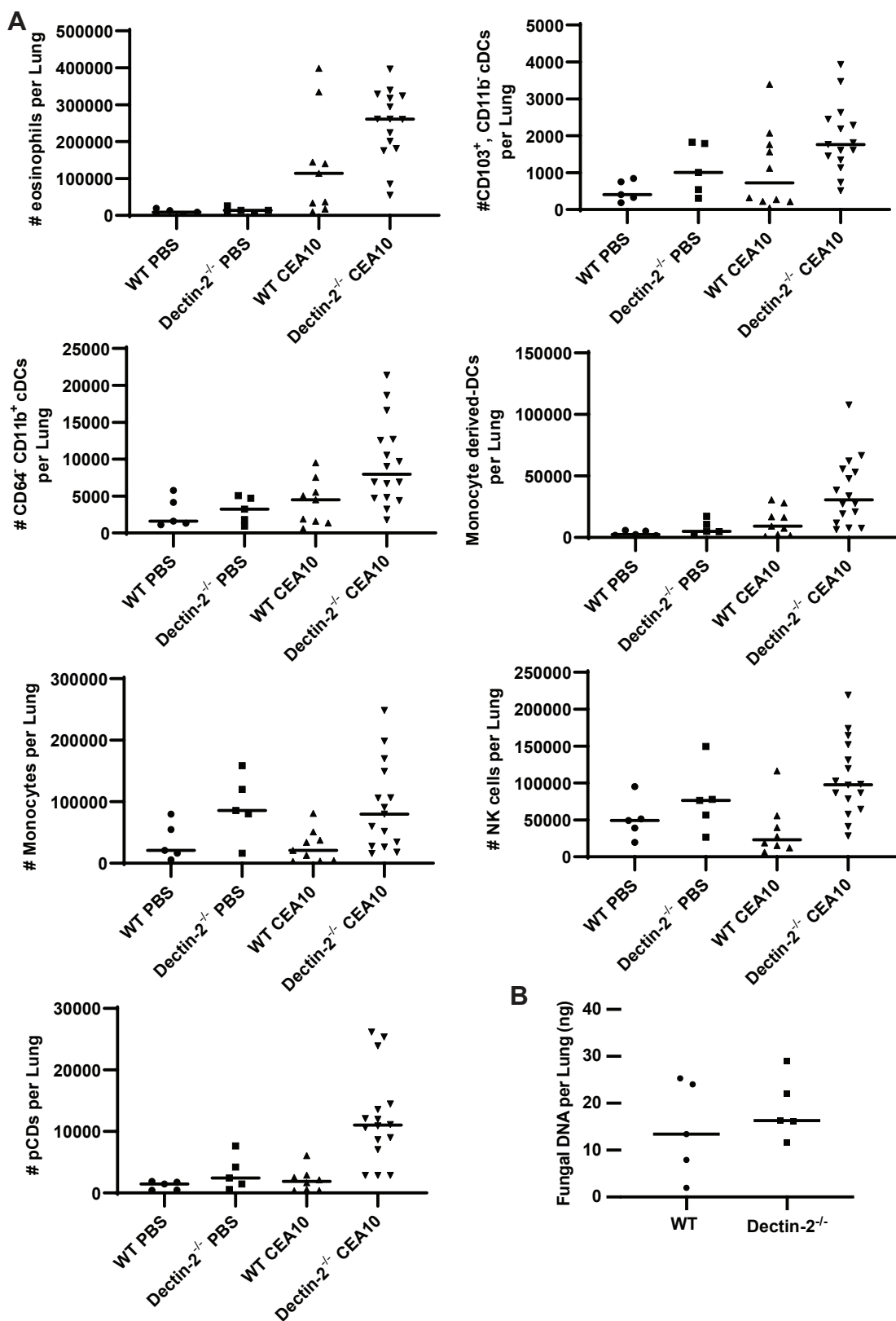

Supplemental Figure S4

Supplement: FIG S4 [file mbio.03184-22-s0006.pdf]

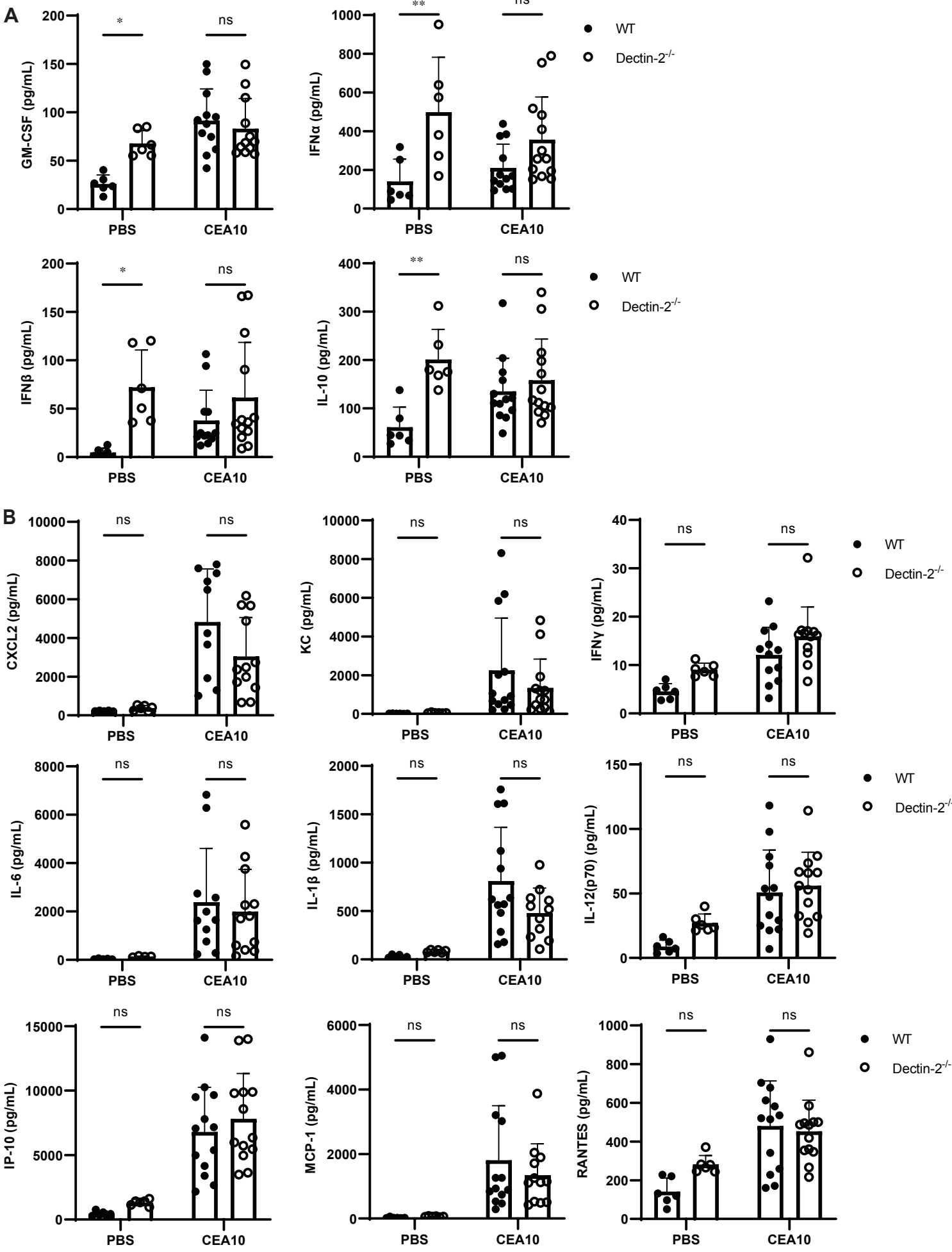

Supplemental Figure S5

Supplement: FIG S5 [file mbio.03184-22-s0007.pdf]
