# Supplementary material for: The C-Type Lectin Receptor Dectin-2 Is a Receptor for Aspergillus fumigatus Galactomannan
Source: mBio. 2023 Jan 4;14(1):e03184-22. doi: 10.1128/mbio.03184-22 (PMC9973300; doi:10.1128/mbio.03184-22)
Supplement: TABLE S1 [file mbio.03184-22-s0002.docx]

**Table S1: List of Differentially Expressed Genes from Nanostring Analysis**

|  | **Dectin-2^OE^**  **Unstim vs GALM** | | **Wild-type**  **Unstim vs GALM** | | **WT vs Dectin-2^OE^**  **(Comparison of log2 Fold Change)** | **Gene Function** |
| --- | --- | --- | --- | --- | --- | --- |
| **Gene ID** | **log2 Fold Change** | **adj.**  ***p*-value^a^** | **log2 Fold Change** | **adj.**  ***p*-value^a^** | ***p*-value ^b^** |  |
| **IL1RN** | 1.96 | ***0.000345*** | 0.721 | 0.0764 | **0.008398** | IL-1 Signaling |
| **RELB** | 1.37 | ***0.0262*** | 0.84 | 0.222 | 0.333463 | Glycan Sensing, Myeloid Activation, NF-κB Signaling |
| **CCL12** | 1.24 | ***0.0133*** | 0.0687 | 0.961 | 0.051755 | Chemokine Signaling, Mononuclear Cell Migration, NLR Signaling, TNF Signaling |
| **MARCO** | 1.11 | ***0.0138*** | 0.259 | 0.646 | 0.064048 | Phagocytosis |
| **CCL4** | 1.07 | ***0.00801*** | 0.751 | 0.0813 | 0.322459 | Chemokine Signaling, DNA Sensing, Mononuclear Cell Migration, NF-κB Signaling |
| **DDIT3** | 0.983 | ***0.0359*** | 0.956 | 0.0866 | 0.945821 | Apoptosis, MAPK Signaling, Proteotoxic Stress |
| **C3** | 0.956 | ***0.000387*** | 0.42 | 0.0526 | **0.014351** | Complement System, Myeloid Activation |
| **CCL3** | 0.897 | ***.0000475*** | 0.502 | ***0.00204*** | **0.005335** | Chemokine Signaling, Mononuclear Cell Migration, Myeloid Activation |
| **HMOX1** | 0.811 | ***0.000308*** | 0.39 | 0.0349 | **0.014440** | HIF1A Signaling, Myeloid Activation, Oxidative Stress Response |
| **TLR2** | 0.805 | ***0.00184*** | -0.0955 | 0.725 | **0.003866** | Host Defense Peptides, Myeloid Activation, TLR Signaling, Virus-Host Interaction |
| **IKBKE** | 0.781 | ***0.00562*** | 0.0605 | 0.876 | **0.016398** | DNA Sensing, NLR Signaling, RNA Sensing, TLR Signaling, Type I Interferon Signaling |
| **PLAU** | 0.777 | ***0.000181*** | 0.619 | ***0.00204*** | 0.122027 | Coagulation, Complement System, Myeloid Activation, NF-κB Signaling |
| **CCL2** | 0.773 | ***0.000239*** | 0.197 | 0.141 | **0.003217** | Chemokine Signaling, Mononuclear Cell Migration, NF-κB Signaling |
| **PLAUR** | 0.743 | ***0.00317*** | 0.0837 | 0.783 | **0.013283** | Coagulation, Complement System, Myeloid Activation |
| **TNF** | 0.695 | ***0.0455*** | 0.00471 | 0.998 | 0.070233 | Mononuclear Cell Migration, Myeloid Activation, NF-κB Signaling, NLR Signaling, TH17 Differentiation, TNF Signaling |
| **CCL7** | 0.664 | ***0.00476*** | 0.299 | 0.169 | 0.071605 | Chemokine Signaling, Mononuclear Cell Migration |
| **PIK3CB** | 0.661 | ***0.00317*** | 0.0933 | 0.709 | ***0.014747*** | BCR Signaling, TNF Signaling |
| **ACSL1** | 0.623 | ***0.00697*** | 0.139 | 0.565 | ***0.036815*** | PPAR Signaling |
| **NFKBIA** | 0.605 | ***0.0213*** | -0.175 | 0.579 | ***0.018616*** | NF-κB Signaling |
| **TLR7** | -0.595 | ***0.00801*** | 0.111 | 0.701 | ***0.011184*** | Myeloid Activation, TLR Signaling, Type I Interferon Signaling |
| **AKT3** | -0.772 | ***0.0194*** | -0.161 | 0.722 | 0.070083 | TNF Signaling |
| **ULK2** | -0.921 | ***0.0379*** | -0.458 | 0.383 | 0.265312 | Autophagy |
| **RNASEL** | -0.929 | ***0.00116*** | -0.315 | 0.171 | ***0.017895*** | Interferon Response Genes, NLR Signaling, Type I Interferon Signaling |
| **FCGRT** | -1.01 | ***0.0318*** | -0.443 | 0.414 | 0.202079 | Myeloid Inflammation |
| **LTC4S** | -1.04 | ***0.0492*** | -0.492 | 0.438 | 0.272114 | Leukotriene and Prostaglandin Inflammation |
| **CXCR4** | -1.37 | ***0.00101*** | -0.112 | 0.811 | ***0.004785*** | Chemokine Signaling, Lymphocyte Trafficking, Virus-Host Interaction |

^a^ Adjusted p-value determined using nCounter Advanced Analysis software using Benjamini-Hochberg False Discovery Rate of 5%.

^b^ Results of unpaired T-test comparing log­­2 Fold Change values between Dectin-2^OE^ and Wild-type using log2 Fold Change and Standard Errors Caluclated using nCounter Advanced Analysis software. T-test Analysis performed using GraphPadPRISM 9
