# Supplementary material for: The C-Type Lectin Receptor Dectin-2 Is a Receptor for Aspergillus fumigatus Galactomannan
Source: mBio. 2023 Jan 4;14(1):e03184-22. doi: 10.1128/mbio.03184-22 (PMC9973300; doi:10.1128/mbio.03184-22)
Supplement: TEXT S1 [file mbio.03184-22-s0001.pdf]

Supplemental Methods:

Generation and quality control of FLP

Carbohydrate coated-FLP consisting of purified β-1,3 glucan (Wellmune, Biothera, *Saccharomyces cerevisiae*, Eagan, MN) and mannan (*S. cerevisiae*, Sigma-Aldrich, M704-1G) were creating using previously published methods (1). Using purified *Aspergillus fumigatus* galactomannan, we created both adsorbed FLP in which the carbohydrate is attached to the bead through electrostatic interactions and covalently-attached FLP using 1,1’-carbonyldiimidazole (CDI)-chemistry as previously published (1). Briefly, 300 mL of amine-coated polystyrene beads (Polysciences, Warrington, PA) were washed three times in sterile anhydrous dimethylsulfoxide (DMSO, Millipore Sigma, Burlington, MA) using 0.45 μm PTFE centrifugal filters (Millipore Sigma, Burlington, MA) to remove any aqueous solution. The beads were then resuspended in either DMSO (adsorbed beads) or DMSO containing 0.5 M 1,1’-carbonyldiimidazole (CDI) and incubated at room temperature for 1 hour with shaking. Beads were then rinsed with DMSO and resuspended in 1mg/mL purified *A. fumigatus* galactomannan in DMSO. Unmodified beads were resuspended just in DMSO without additional carbohydrates. The mixture was incubated for 1 hour at room temperature with shaking and centrifuged to remove the DMSO and excess carbohydrates. Beads were then washed, and resuspended in PBS, counted on a Luna™ automated cell counter (Logos Biosystems, Annandale, VA), and stored at 4°C.

To confirm the association of carbohydrates with the FLPs and ensure rigor, each newly created batch of FLPs was analyzed by flow cytometry. Briefly, 3 mL of

beads were blocked overnight in PBS containing 2% BSA at 4℃. To analyze mannan binding to the surface, 10 mL of 20 mg/mL stock fluorescein labeled Concanavalin A (Con A; Invitrogen, Thermo Fisher Scientific, Carlsbad, CA; C827) was added to each sample and incubated for 1 hour at room temperature while shaking. Samples were washed and resuspended in FACS buffer (PBS containing 2% BSA) and then analyzed on a BD FACSCalibur^{TM} or BD FACSCelesta^{TM} Flow Cytometer (BD, Franklin Lakes, NJ). For analysis of β-1,3 glucan, 20 ng of primary monoclonal anti-β-1,3 glucan antibody (Biosupplies Australia Pty LTD, Victoria, Australia; 400-2) was added to each sample and incubated for 1 hour at room temperature. FLPs were washed and then incubated for 30 minutes with a 1:100 dilution of secondary rabbit anti-mouse IgG conjugated with Alexa Fluor 488 (Invitrogen, Carlsbad, CA; A11059). FLPs were then washed, resuspended in FACS buffer, and analyzed by flow cytometry. For galactomannan-coated beads, FLPs were centrifuged, and the blocking buffer was removed. FLPs were resuspended in 50 ml of component R6 from the Platelia *Aspergillus* Ag kit (Bio-Rad, Hercules, CA) containing rat anti-galactomannan IgM antibody and were incubated for 1 hour at room temperature. FLPs were then washed and resuspended in a 1:200 dilution of secondary goat anti-rat IgM conjugated with Alexa Fluor 488 (Invitrogen, Carlsbad, CA; A-21212) in FACs Buffer. FLPs were incubated for 30 minutes at room temperature and then washed and resuspended in FACS buffer.

To assess the stability of carbohydrate association, 10 mL of FLP were incubated in 800 mL of either 3% SDS, 1X NP40, 1% Triton X-100 for 1 hour at room temperature with constant shaking, or 3% SDS at 95^0C for 1 hour. The FLPs were centrifuged,

washed with detergent, and washed three times with FACS buffer. FLPs were then subjected to antibody staining to assess for carbohydrate association as detailed above.

For fluorescent imaging of carbohydrate coated FLPs, FLPs were fluorescently labeled in the same manner as for flow cytometry and then placed onto glass slides. FLPs were imaged using a Nikon Ti-E inverted microscope with a CSU-X1 confocal spinning-disk head (Yokogawa, Sugarland, TX) with a Coherent 4-Watt laser (Coherent, Santa Clara, CA) as the excitation source. Images were acquired through an EM-CCD camera (Hamamatsu, C9100-13, Bridgewater, NJ) and performed using MetaMorph® software (Molecular Devices, Downington, PA). Image data files were processed using Adobe Photoshop 2021 and assembled in Adobe Illustrator 2022 (Adobe Systems, San Jose, CA).

Lentiviral transduction of cells with Dectin-2 and FcRγ

Murine Dectin-2 was amplified from plasmid pFB-dectin2-IRES-eGFP from Caetano Reis e Sousa (London Research Institute, London, U.K.) using a 5' primer containing NotI digestion site with sequence TATTGCGGCCGCCATGGTGCAGGAAAGACAATCCCAAGG and a 3' primer containing a BglII restriction site with sequence TCGACGATAGATCTTCATAGGTAAATCTTCTTCATTTCACATATTGAATTG. Primers were synthesized by Integrated DNA technologies (IDT, Research Triangle Park, NC). The gamma chain protein (FcRγ) of the Fc-gamma receptor (FcγR) was PCR amplified from plasmid pSVL-mo FcεRI gamma (plasmid was a gift from Jean Kinet, Addgene plasmid #8372), using 5' primer containing a NotI digestion site with sequence

TATTGCGGCCGCCATGATCTCAGCCGTGATCTTGTTCTTG and 3’ primer containing a BamHI digestion site TCGACGATGGATCCCTACTGGGGTGGTTTTTCATGCTTC. The constructs were cloned into a pHAGEII lentiviral vector containing either puromycin or blasticidin selectable markers using NotI and BamHI digestion and sequence verified. Generation of lentivirus in HEK293T cells and transduction was performed as previously described (2). Cells were selected for uptake of the lentivirus vector using puromycin or blasticidin at 5 μg/mL. After selection, expression of the desired gene was confirmed using western blot. Surface localization of Dectin-2 was confirmed using flow cytometry using anti-murine Dectin-2-APC (R&D Systems, Minneapolis, MN, FAB1515A) or anti-human Dectin-2 polyclonal goat antibody (R&D, AF3114) with Donkey anti-Goat AF488 secondary antibody (Invitrogen, Thermo Fisher Scientific, Rockford, IL, A11055).

CLR ligand reporter cell assay

Reporter cells were used based on previously published protocols (3). B3Z and BWZ cells carrying an NFAT-lacZ reporter construct have been previously described (4, 5). B3Z cells expressing murine Dectin-2 or a wildtype murine FCRγ-chain as well as BWZ cells expressing murine Dectin-1CD3ζ (the extracellular domain of mouse Dectin-1 fused to CD3ζ intracellular tail) were constructed by Dr. Caetano Reis e Sousa (6). Mincle and Dectin-3 (MCL) expressing cells were created as previously described (3). Briefly, cells were seeded at 1 x 10^5 cells per well in 96-well plates followed by stimulation with FLPs or media alone. Cells were incubated for 18 hours in a tissue culture incubator at 37℃ with 5% CO_2. Cells were pelleted and the supernatant was removed. Cells were lysed with triton lysis buffer (1% Triton X-100, 9 mM KH_2PO_4, 90

mM K_2HPO_4) on ice for 30 minutes. Plates were centrifuged to pellet cellular debris and 40 mL of lysate was transferred to a new 96-well plate. Assay buffer containing chlorophenol red-β-D-galactopyranoside (CPRG) was added to each well and plates were incubated for 6 hours at 37℃ protected from light and then absorbance at 560 nm and 620 nm was measured using an i3x Spectrophotometer (Molecular Devices LLC, San Jose, CA).

Fungal Fc Protein Binding Assays

A. fumigatus resting conidia, swollen conidia and early germlings were grown as previously described in 8-well Nunc™Lab-Tek® II Chambered Coverglass (Thermo Fisher Scientific, Rockford, IL). Fungi was washed with PBS and then blocked by incubated in PBS containing 1% BSA for 1 hour at 4^0C. *Aspergillus* was then washed and resuspended in 200 µL of binding buffer (20 mM Tris-HCL, pH 7.4, 150 mM NaCl, 10 mM $CaCl_2$, 0.05% Tween-20). Dectin-2(murine)- IgG1 Fc (human) fusion protein (ENZO Life Sciences, Inc, Farmingdale, NY) or IgG1 Fc (human) protein (Thermo Fisher Scientific, Rockford, IL) was added to a final concentration of 10 µg/mL and samples were incubated with gentle mixing for 1 hours at 4^0C. Following washing, samples were resuspended in binding buffer containing a 1:500 dilution of anti-human-IgG1-Fc conjugated to Alexa Fluor 647 (BioLegend, San Diego, CA; 410713) and incubated for 30 minutes at 4^0C in the dark. Samples were washed and resuspended in binding buffer and were imaged using a Nikon Ti-E inverted microscope with a CSU-X1 confocal spinning-disk head (Yokogawa, Sugarland, TX) with a Coherent 4-Watt laser (Coherent, Santa Clara, CA) as the excitation source. Images were acquired through

an EM-CCD camera (Hamamatsu, C9100-13, Bridgewater, NJ) and using MetaMorph® software (Molecular Devices, Downington, PA). Image data files were processed using Adobe Photoshop 2021 and assembled in Adobe Illustrator 2022 (Adobe Systems, San Jose, CA).

Western blot

Cells were placed on ice and lysed with 1% NP40 lysis buffer containing protease and phosphatase inhibitors. Proteins were denatured using NuPAGE LDS Sample Buffer (Thermo Fisher Scientific, Rockford, IL). The proteins were resolved by SDS-PAGE on 4-12% NuPAGE gels using NuPAGE MOPS buffer (NuPage gels, Thermo Fisher Scientific, Rockford, IL), and transferred to methanol-activated PVDF membrane (Perkin Elmer, Waltham, MA) using transfer buffer (0.025M Tris, 0.192 M glycine, 20% methanol) and electrophoretic transfer at 100V for 1 hour. For detection of proteins, PVDF membranes were blocked for 1 hour at room temperature in 5% BSA in PBS-0.01% Tween 20 (PBST). Blots were incubated with anti-phospho spleen tyrosine kinase (Syk) antibody (Cell Signaling Technologies, Danvers, MA; 2710S) or anti-total Syk antibody (Cell Signaling Technologies, 13198S), in 5% BSA in PBST overnight at 4℃. The membranes were washed and incubated with secondary swine anti-rabbit HRP conjugated antibody (Agilent DAKO, Santa Clara, CA, P0399) in 1% milk in PBST for 1 hour at room temperature. Membranes were washed and then visualized using Western Lightning Plus ECL chemiluminescent substrate (Perkin Elmer, Waltham, MA) on Kodak BioMax XAR film (MilliporeSigma, Burlington, MA). Films were then scanned and

processed using Adobe Photoshop 2021. Any contrast adjustments were applied evenly to the entire image and adheres to standards set forth by the scientific community (7).

Transcriptional Analysis

Wildtype and Dectin-2 expressing immortalized C57BL/6 macrophages were plated at 150,000 cells / well in 12 well cell-culture plates in complete RPMI media. Macrophages were either unstimulated or stimulated with Galactomannan FLP at a target to effector ration of 30:1 for 6 hours. Following stimulation, media was removed from the wells and the cells were lysed with Qiagen RLT Buffer (Qiagen, Germantown, MD) containing β-mercaptoethanol, passed through a QIAshredder Column and RNA isolated using the Qiagen RNeasy Kit according to the manufacturers instructions. Concentration of the RNA was quantified using a NanoDrop™ One Microvolume UV-Vis Spectrophotometer (Thermo Fisher Scientific, Rockford, IL).

NanoString (nanoString, Seattle, WA) profiling of transcriptional responses was performed using the nCounter® Host Response Panel according to manufacturers instructions. Total RNA (25 ng) was used for hybridization reactions at 65^0C for 24 hours. The hybridized samples were loaded on the nCounter® Cartridge and analyzed using a nCounter® Sprint Profiler. The resulting data was analyzed using the nSolver analysis software. Negative controls were used for background subtraction. Positive cartridge controls as well as housekeeping genes (with counts greater than 100) were used for data normalization. The nSolver® Advance Analysis 2.0 was used to analyze gene expression data for the following comparisons: Dectin-2^{OE} macrophages stimulated with GALM FLP versus baseline of unstimulated and Wild -type macrophages stimulated with GALM FLP versus baseline of unstimulated. Genes with counts < 20 were excluded from the analysis, leaving a total of 443 out of 785 genes in the final analysis. Fold changes and p-values were calculated using the fast/recommended method as described in the nCounter® Advanced Analysis 2.0 User Manual.

Adjusted p-values were obtained using the Benjamini-Hochberg method of estimating false discovery rates. Genes were considered to be significant if they had a > +/- 1.5 fold change in expression and had an adjusted p-value <0.5. To compare the Log_2 Fold Change for genes between Dectin-2^{OE} macrophages and Wild-type macrophages, multiple unpaired t-tests were performed using PRISM 9 Software (GraphPad Software, San Diego, CA) using the Log_2 Fold Change and Standard Error for the differentially expressed genes generated by the nSolver® Advance Analysis 2.0 software.

Cytokine Quantification

1 x10^5 murine macrophages (immortalized or BMDM) were plated in triplicate in tissue culture treated 48-well plates and stimulated overnight with FLP or fungal germlings at the indicated target-to-effector ratios. Supernatants were collected for cytokine analysis using ELISA (Duoset, R&D Systems, Minneapolis, MN) and read using an i3X Spectrophotometer (Molecular Devices, LLC, San Jose, CA). Results were analyzed using PRISM9 software (GraphPad Software, San Diego, CA).

To quantify pulmonary cytokines, mouse lungs were dissected at the indicated time post-infection with either PBS or *A. fumigatus* strain CEA10. Lungs were removed and placed into 2 mL of PBS and homogenized (Omni Tissue Homogenizer, Omni International, Kennesaw, GA) for 2 minutes each and then frozen at -80^0C. At the time of analysis, lung homogenates were thawed and then centrifuged at 13,000 rpm x 10 minutes to pellet cellular debris. Supernatants were used for cytokine analysis using the Biolegend® LEGENDplex™ Mouse Anti-virus Response Panel containing (IFN-γ, CXCL1 (KC), TNFα, CCL2 (MCP-1), IL-12p70, CCL5 (RANTES), IL-1β, IP-10 (CXCL10), GM-CSF, IL-10, IFN-β, IFN-α, IL-6) according to the manufacturers instructions and analyzed using the LEGENDplex™ Data Analysis Software Suite. CXCL2 concentration was analyzed using ELISA (Duoset, R&D Systems, Minneapolis, MN) and read using an i3X Spectrophotometer (Molecular Devices, LLC, San Jose, CA). TNFα

concentrations were confirmed using ELISA (Duoset, R&D Systems, Minneapolis, MN). Results were analyzed using PRISM9 software (GraphPad Software, San Diego, CA).

Immunophenotyping of pulmonary infiltrates

Wildtype or Dectin-$2^{-/-}$ mice were infected with 4 x 10^7 conidia of *A. fumigatus* strain CEA10 through oropharyngeal inhalation. Each experiment consisted of at least 7 wild-type and 7 Dectin-$2^{-/-}$ mice infected with CEA10 *Aspergillus* and 5 mock infection (PBS only) control per mouse line. Mice were euthanized at 48 hours post-infection and immunophenotyping was performed as previously described(8). Briefly, lungs were perfused with PBS through cardiac puncture to flush intravascular cells. Luns were excised, placed into Digestion Solution (38 mg Collagenase Type I and 100 U DNase I in 100 ml RPMI 1640), finely minced using scissors and incubated for 1 hour with shaking at 37℃. After 1 hour, 800 ml of 0.5 M EDTA was added to each sample to stop the collagenase activity. Lungs samples were passed through a 70 mM filter, pelleted, resuspended in a 40% Percoll-RPMI solution, and under-layered with a 67% Percoll-RPMI solution. Samples were centrifuged at 650 x g for 20 minutes and leukocytes were collected at the interface between layers and washed twice with FACS buffer (PBS with 2% FBS). Cells were counted and 1 – 2 x 10^6 cells were stained for flow cytometry analysis. Fc block (anti-Mouse CD16/CD32, Thermo Fisher Scientific, Rockford, IL, 14-0161-85) was added to cells at 1:100 dilution and incubated at room temperature for 15 minutes. Cells were then labeled with a mixture of the following fluorophore-conjugated antibodies for 1 hour at 4℃ (all antibodies are from Biolegend, San Diego, CA unless otherwise noted): anti-CD45 BV605 (Biolegend, 103139), anti-CD90.2 BV786 (Biolegend, 105331), anti-CD19 PE-Dazzle (Biolegend, 115553), anti-CD4 BV510 (Biolegend, 100559), anti-CD8 APC FIRE (Biolegend, 100765), anti-Siglec-F APC (Biolegend, 155507), anti-Ly6G AF 488 (Biolegend, 127625), anti-Ly6C BV510 (Biolegend, 128033), anti-I-A/I-E AF700 (Biolegend, 107622), anti-NCR1 BV421 (Biolegend, 137611), anti-CD64 PE (Biolegend, 139303), anti-CD103 BV421 (Biolegend,

121421), anti-CD11b APC FIRE (Biolegend, 101261), anti-CD11c BV650 (Biolegend, 117339), anti-B220/CD45R PE (Biolegend, 103207), anti-TCRγδ APC (Biolegend, 118115), and anti-TCRβ BV650 (Biolegend, 109251). Cells were washed with FACS buffer, and 7-AAD (Stem Cell Technologies, 75001.1) was added for live-dead cell staining. Flow cytometry was performed using BD FACSCelesta™ and analysis was performed using FlowJo 10 software (BD, Ashland, OR). All cells were first gated based on the following parameters : 7-AAD^{lo}, CD45^{+}, CD90.2^{-}, CD19^{-} and then were further gated using a previously described gating strategy to identify discrete immune cell populations (8).

Quantification of Fungal Burden in Lungs

Wildtype or Dectin-2$^{-/-}$ mice were infected with 4 x 10^7 conidia of *A. fumigatus* strain CEA10 through oropharyngeal inhalation under isoflurane anesthesia**.** Each experiment consisted of 5 wild-type and 5 Dectin-2$^{-/-}$ mice infected with CEA10 *Aspergillus* and one mock infection (PBS only) control per mouse line. After 48 hours lungs were extracted, frozen at -80°C, and lyophilized overnight. Freeze-dried tissue was homogenized using 0.5 mm glass beads with a TissueLyser LT homogenizer (Qiagen, Germantown, MD) to create a fine powder. DNA was extracted from the homogenized tissue using the E.Z.N.A Fungal DNA Extraction Kit (Omega Bio-tek, Norcross, GA). The total quantity of DNA extracted from each sample was quantified using a NanoDrop™ One Microvolume UV-Vis Spectrophotometer (Thermo Fisher Scientific, Rockford, IL). Fungal DNA in each sample was quantified using qPCR. A standard curve was created using *A. fumigatus* genomic DNA and consisted of 10-fold serial dilutions from 1 pg to 100 ng. 500 ng of sample DNA was analyzed per reaction and each sample was analyzed in triplicate. The sequences for the amplification primers and dual-labeled fluorogenic hybridization probe for the *A. fumigatus* 18S rRNA gene were previously published(9). All three probes were synthesized by Integrated DNA Technologies (IDT, Research Triangle Park, NC).

Each 20 μL qPCR contained TaqMan® Fast Advanced Master Mix (Thermo Fisher Scientific, Rockland, IL), 500 ng of sample DNA and 1 μM each of forward primer, reverse primer and probe. Samples were run and analyzed using the Applied Biosystems™ 7500 Fast Real-Time PCR System (Thermo Fisher Scientific, Rockland, IL). The percent of fungal DNA in each sample was determined by dividing the quantified fungal DNA in each PCR by 500 ng (input DNA per sample). The total DNA obtained from each lung sample was multiplied by the percent fungal DNA to obtain the total fungal DNA/lung.

Colony Forming Unit (CFU)

Wildtype or Dectin-$2^{-/-}$ mice were infected with 4 x 10^7 conidia of *A. fumigatus* strain CEA10 through oropharyngeal inhalation under isoflurane anesthesia. Each experiment consisted of 5 wild-type and 5 Dectin-$2^{-/-}$ mice infected with CEA10 *Aspergillus* and one mock infection (PBS only) control per mouse line. After 48 hours lungs were extracted and placed into conical tubes containing sterile PBS. The weight of each tube pre- and post- lung addition was recorded to determine the weight of the tissue. The lungs were homogenized (Omni Tissue Homogenizer, Omni International, Kennesaw, GA) for 2 minutes each and then serial diultions were performed and plates onto SBD agar. Plates were incubated for 48 hours at 30^0C and colonies were counted. Data are reported at the number of colony forming units per gram of tissue. There was no growth from PBS only control mice.

In vivo Aspergillus survival model

Wild-type C57BL/6 mice or Dectin-$2^{-/-}$ mice were immunosuppressed with 40 mg/kg subcutaneous triamcinolone acetonide (Kenalog®-10, Bristol-Myers Squibb, New York, NY) starting the day prior to infection and every 7 days thereafter. The mice were infected with 2.4 x10^4 CEA10 conidia intranasally under isoflurane anesthesia. For each

experiment 10 mice were infected with CEA10 and 3 mice were treated with PBS only per genotype. Mice were monitored twice daily for the first 5 days of the experiment and then daily thereafter. Mice were assessed based upon a 12-point scale to determine when an individual animal should be euthanized due to illness and removed from the study. This was developed in conjunction with the MGH IACUC and includes the following symptoms and points assigned to each in parentheses: hunched posture (3), ruffled and/or matted fur (3), shivering (3), abnormal breathing (increased respiratory rate) (12), 75% reduction in activity compared to controls (3), inactivity leading to inability to acquire food or water (12), and barrel rolling (12). If any animal reaches 12 points, they are humanely euthanized and counted as a death for the purposes of survival analysis. All animal experiments were approved under the MGH IACUC protocol #2008N000078.

SUPPLEMENTAL REFERENCES

1. Tam JM, Mansour MK, Khan NS, Yoder NC, Vyas JM. 2012. Use of fungal derived polysaccharide-conjugated particles to probe Dectin-1 responses in innate immunity. Integr Biol (Camb) 4:220-7.
2. Mansour MK, Tam JM, Khan NS, Seward M, Davids PJ, Puranam S, Sokolovska A, Sykes DB, Dagher Z, Becker C, Tanne A, Reedy JL, Stuart LM, Vyas JM. 2013. Dectin-1 activation controls maturation of beta-1,3-glucan-containing phagosomes. J Biol Chem 288:16043-54.
3. Wang H, LeBert V, Hung CY, Galles K, Saijo S, Lin X, Cole GT, Klein BS, Wüthrich M. 2014. C-type lectin receptors differentially induce th17 cells and

vaccine immunity to the endemic mycosis of North America. J Immunol 192:1107-19.

4. Karttunen J, Sanderson S, Shastri N. 1992. Detection of rare antigen-presenting cells by the lacZ T-cell activation assay suggests an expression cloning strategy for T-cell antigens. Proc Natl Acad Sci U S A 89:6020-4.

5. Fiering S, Northrop JP, Nolan GP, Mattila PS, Crabtree GR, Herzenberg LA. 1990. Single cell assay of a transcription factor reveals a threshold in transcription activated by signals emanating from the T-cell antigen receptor. Genes Dev 4:1823-34.

6. Robinson MJ, Osorio F, Rosas M, Freitas RP, Schweighoffer E, Gross O, Verbeek JS, Ruland J, Tybulewicz V, Brown GD, Moita LF, Taylor PR, Reis e Sousa C. 2009. Dectin-2 is a Syk-coupled pattern recognition receptor crucial for Th17 responses to fungal infection. J Exp Med 206:2037-51.

7. Rossner M, Yamada KM. 2004. What's in a picture? The temptation of image manipulation. J Cell Biol 166:11-5.

8. Wiesner DL, Merkhofer RM, Ober C, Kujoth GC, Niu M, Keller NP, Gern JE, Brockman-Schneider RA, Evans MD, Jackson DJ, Warner T, Jarjour NN, Esnault SJ, Feldman MB, Freeman M, Mou H, Vyas JM, Klein BS. 2020. Club Cell TRPV4 Serves as a Damage Sensor Driving Lung Allergic Inflammation. Cell Host Microbe 27:614-628 e6.

9. Bowman JC, Abruzzo GK, Anderson JW, Flattery AM, Gill CJ, Pikounis VB, Schmatz DM, Liberator PA, Douglas CM. 2001. Quantitative PCR assay to measure *Aspergillus fumigatus* burden in a murine model of disseminated

aspergillosis: demonstration of efficacy of caspofungin acetate. Antimicrob Agents Chemother 45:3474-81.
